# Early activation of Virchow’s triad in feline hypertrophic cardiomyopathy: beyond spontaneous echo contrast and insights into translational thromboembolism

**DOI:** 10.3389/fvets.2026.1870206

**Published:** 2026-07-02

**Authors:** Felipe Gaia de Sousa, Ruthnea Aparecida Lázaro Muzzi, Roberto Baracat de Araújo, Rafael Resende Faleiros, Suzane Lilian Beier

**Affiliations:** 1 Department of Veterinary Clinic and Surgery, Veterinary School, Federal University of Minas Gerais – UFMG, Belo Horizonte, Minas Gerais, Brazil; 2 VETHEART – Cardiovascular Physiology and Veterinary Cardiology Research Group, Belo Horizonte, Minas Gerais, Brazil; 3 National Institute of Science and Technology in Nanobiopharmaceutics – INCT NanoBiofar, Belo Horizonte, Minas Gerais, Brazil; 4 Department of Veterinary Medicine, Faculty of Animal Science and Veterinary Medicine, Federal University of Lavras – FZMV/UFLA, Lavras, Minas Gerais, Brazil

**Keywords:** blood stasis, coagulation, endothelial injury, prognosis, thrombogenesis

## Abstract

The hypertrophic cardiomyopathy (HCM) phenotype is closely associated with arterial thromboembolism (ATE). The pathophysiology of ATE is linked to activation of Virchow’s triad, characterized by endothelial injury, blood stasis, and hypercoagulability. Together, these factors create a prothrombotic microenvironment that may precede overt clinical manifestations. Although spontaneous echocardiographic contrast (SEC) is widely used in clinical practice as a marker of thromboembolic risk, it should be regarded as a late finding. This review explores the early activation of Virchow’s triad in cats with HCM phenotype, with emphasis on atrial remodeling, hemodynamic alterations, and endothelial dysfunction preceding detectable SEC. Evidence suggests that changes in left atrial appendage dynamics contribute to localized stasis, while systemic mechanisms, including platelet activation, thrombin generation, and inflammatory processes, promote a hypercoagulable state. Additionally, the presence of SEC should be interpreted as a biomarker of blood stasis rather than a causal factor. Reliance on SEC for thrombotic risk assessment reflects limitations in identifying the pre-thrombotic state, particularly due to the lack of standardized biomarkers for routine clinical use. Therefore, improved thromboembolic risk stratification in cats with HCM phenotype is needed. Overall, thrombogenesis in cats with cardiac disease is a dynamic, multifactorial, and progressive process that begins prior to detectable echocardiographic changes. A clearer understanding of these mechanisms may enable earlier identification of high-risk patients and support preventive therapeutic strategies. This review analyzes the early activation of Virchow’s triad in cats with HCM phenotype and its relationship with atrial thrombus formation, with emphasis on thrombogenic mechanisms preceding SEC and their implications for ATE.

## 1 Introduction

Among the causes frequently associated with arterial thromboembolism (ATE) in cats, the hypertrophic cardiomyopathy (HCM) phenotype is commonly reported. The HCM phenotype is characterized by myocardial hypertrophy, either symmetric or asymmetric, affecting the left ventricular (LV) free wall and/or the interventricular septum (1–8). Asymmetric forms of HCM are the most common and are primarily associated with the left ventricular outflow tract (LVOT), characterizing an obstructive presentation (LVOTO) (2, 9, 10). With disease progression, systolic anterior motion (SAM) of the mitral valve leaflet toward the LVOT may occur, further exacerbating the clinical condition (1, 2, 11–13). Affected cats may present with subclinical disease (stage B) or clinical signs (stage C or D) of congestive heart failure (CHF) (1–6, 8, 14–20).

**Figure 1:**
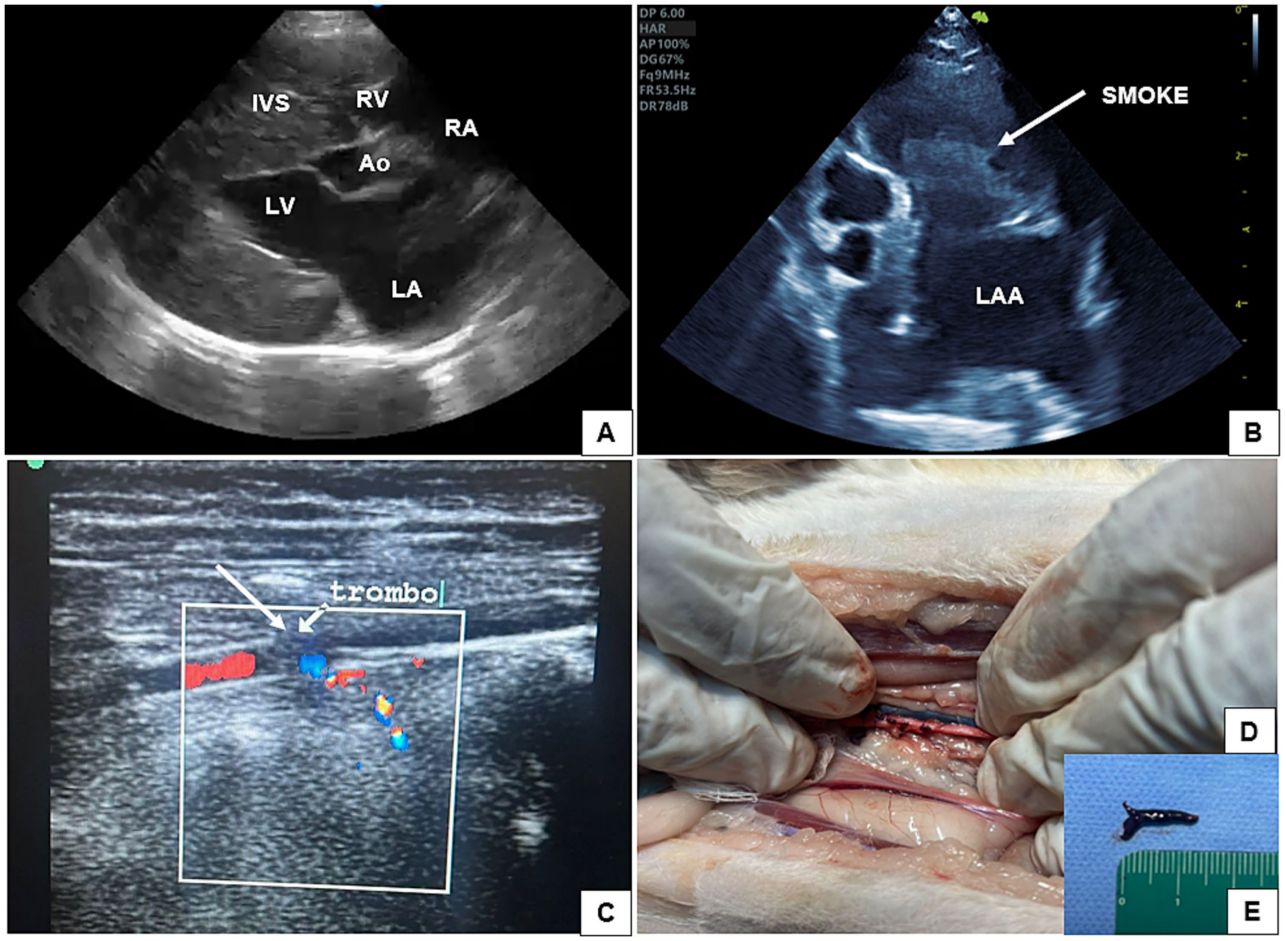
**(A)** Hypertrophic cardiomyopathy phenotype; parasternal long-axis, five-chamber view (Courtesy of DVM Giovanni Canta). **(B)** Feline auricle showing the presence of *smoke* (arrow) in a patient with cardiomyopathy (Courtesy of DVM André Luiz Alegre). **(C)** Doppler ultrasonographic evaluation showing the presence of a thrombus (arrow) and flow obstruction (Courtesy of DVM José Neto Cruz and Guilherme Chaves). **(D)** Surgical thrombectomy procedure in a cat with feline arterial thromboembolism (Courtesy of DVM José Neto Cruz). **(E)** Thrombus removed by thrombectomy procedure in a cat (Courtesy of DVM José Neto Cruz). RA, right atrium; RV, right ventricle; LA, left atrium; LV, left ventricle; Ao, aorta; IVS, interventricular septum.

ATE is a condition associated with HCM phenotype and develops in the presence of factors that predispose to thrombus formation (1–3) (Figure 1). The main mechanism underlying ATE is the activation of Virchow’s triad, which is frequently observed in cats with HCM (21–25). It has been proposed that cats with HCM may exhibit a pre-thrombotic state, although direct evidence in cats remains limited (Figure 1). Clinical manifestations of ATE vary depending on thrombus characteristics, location, and duration of occlusion (21, 26) (Figure 1). Affected cats often present with acute clinical signs of severe pain and marked perfusion deficits (21, 26–28).

Cats with ATE exhibit the five P’s: “pallor (i.e., purple, cyanotic or pale toes), polar/poikilothermy (ie, cold extremities), pulselessness, paralysis/paresis and pain” (21). Furthermore, ATE may occur unilaterally or bilaterally and is often associated with rapid clinical deterioration (21, 29). Bilateral presentations typically originate at the aortic bifurcation, leading to severe perfusion impairment and, in some cases, limb loss (21, 24, 29). Notably, these clinical manifestations may be easily mistaken for neurological disorders due to overlapping signs.

Spontaneous echocardiographic contrast (SEC), also referred to as “smoke,” is commonly recognized within the left atrium (LA) as a clinical marker associated with blood stasis, atrial dysfunction, and increased thromboembolic risk (1, 2, 30–34). However, evidence from feline studies, together with extrapolation from human data, suggests that thrombogenic mechanisms may precede the development of detectable SEC (2). Although the high risk of thromboembolic complications in cats with HCM phenotype is well established, prophylactic strategies (e.g., antiplatelet agents, anticoagulants, and thrombolytic therapy) are recommended in cats considered at increased risk of ATE (21, 26–28). Most of the literature has focused on established markers such as SEC, however, thrombogenic mechanisms remain incompletely understood. This review focuses on early Virchow’s triad activation in cats with HCM phenotype and its implications for thromboembolic risk stratification. Furthermore, it highlights the implications and current limitations for thromboembolic risk stratification in feline HCM, emphasizing the need for an early and mechanism-based approach to risk identification.

## 2 Virchow’s triad applied to HCM phenotype

**Figure 2:**
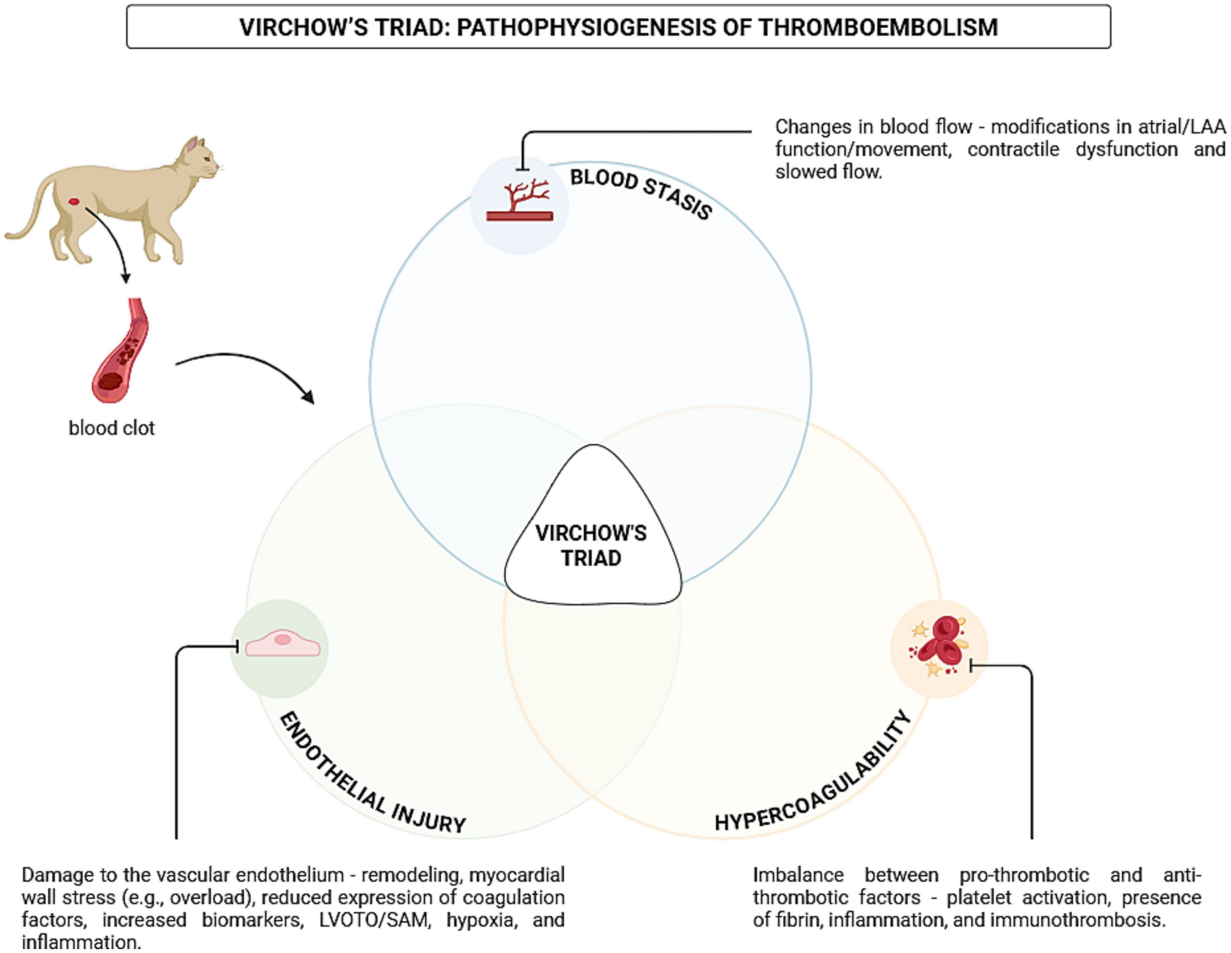
Schematic representation of Virchow’s triad: hypercoagulability, blood stasis, and endothelial injury. LAA, left atrial appendage; LVOTO, left ventricular outflow tract obstruction; SAM, systolic anterior motion of the mitral valve. Created with BioRender.

Virchow’s triad comprises hypercoagulability, blood stasis, and endothelial injury, all contributing to thrombogenesis (35, 36) (Figure 2). Humans and animals may exhibit a prothrombotic state characterized by altered blood flow (e.g., turbulence or stasis) and endothelial activation, which together create a permissive microenvironment for thrombogenesis (21–26, 35, 37–41). According to Donadini et al. (42), thrombus formation may arise from an imbalance between prothrombotic and antithrombotic factors. Under physiological conditions, endothelial cells provide an anticoagulant surface that inhibits thrombus formation, which helps explain why endothelial dysfunction is strongly associated with a prothrombotic state (42). The importance of endothelial dysfunction is well established, and Donadini et al. (42) highlight that “pivotal role of the endothelium in both health and disease”. Therefore, understanding the mechanisms that may trigger progression to active thrombogenesis remains an important area of investigation. Thrombus formation is closely associated with endothelial injury, hemodynamic alterations, platelet activation, and inflammatory pathways, potentially contributing to the development of SEC and thrombogenesis (22–25, 35, 40, 41, 43).

Affected cats, particularly those at stage B1 or beyond, are considered at increased risk for the development of ATE. From a pathophysiological perspective, the HCM phenotype is believed to promote conditions associated with increased thrombotic risk (26). Concentric hypertrophy of the interventricular septum and/or left ventricular free wall reduces the internal diameter of the LV, with compensatory remodeling to maintain adequate volumetric function (1, 2, 4, 16, 19). Asymmetric forms associated with LVOTO and SAM are also associated with atrial remodeling and reduced flow velocities, particularly within the left atrial appendage (LAA) (1, 2, 9, 16, 19, 44) (Figure 2).

Evidence suggests the presence of a hypercoagulable state in some affected cats, involving multiple cellular components such as platelets and erythrocytes (24) (Figure 2). This imbalance shifts hemostatic equilibrium toward coagulation. Platelets play a central role in both hemostasis and coagulation, becoming activated in response to tissue injury and contributing to the formation of fibrin-rich platelet plugs (24). Additional proposed mechanisms include enhanced thrombin generation, inflammatory activation, often reflected by indices such as the neutrophil-to-lymphocyte ratio (NLR), and dysregulation of immunothrombosis pathways (e.g., neutrophil extracellular traps – NETs) (24). Although promising, these pathways remain primarily investigational. Emerging evidence suggests that platelet-related parameters may play an important role in cats with HCM and thromboembolic complications, particularly platelet large cell count (PLCC) and platelet large cell ratio (PLCR) (45). Current evidence highlights the potential role of PLCC and PLCR in mechanisms associated with cardiovascular disease detection, clinical stage progression, and thromboembolic complications (45). These parameters have been proposed as emerging markers associated with early platelet activation, particularly regarding thrombus formation dynamics and early risk identification. However, they still require further validation. Factors such as platelet heterogeneity may be related to platelet activation and thrombogenesis, particularly through changes in morphology and thrombotic potential throughout disease progression (29). Although not frequently described, platelet heterogeneity has been reported as a potentially important factor in cases of ATE.

Blood stasis arises primarily from atrial dilation secondary to remodeling and mechanical dysfunction, as well as reduced blood flow velocity within the LAA, a clinically useful parameter for thromboembolic risk assessment (19, 24) (Figure 2). Endothelial injury, in turn, is associated with structural remodeling and altered expression of coagulation-related factors, including von Willebrand factor (24). Collectively, these alterations provide evidence that cats with the HCM phenotype fulfill the criteria for activation of Virchow’s triad, thereby predisposing to thrombogenesis and ATE (Figure 2).

From a translational perspective, risk factors for ATE in humans and cats share similarities. In humans with HCM, atrial fibrillation, LA enlargement, and CHF are associated with increased thromboembolic risk (46). Similarly, in cats, prior thromboembolic events, LA enlargement, atrial mechanical dysfunction, male sex, intracardiac thrombi, and echocardiographic findings (e.g., SEC) are linked to the development of ATE (24). These parallels suggest that comparative insights between species may provide a valuable framework for improving understanding of ATE and for exploring translational preventive strategies.

### 2.1 Atrial endothelial injury

**Figure 3:**
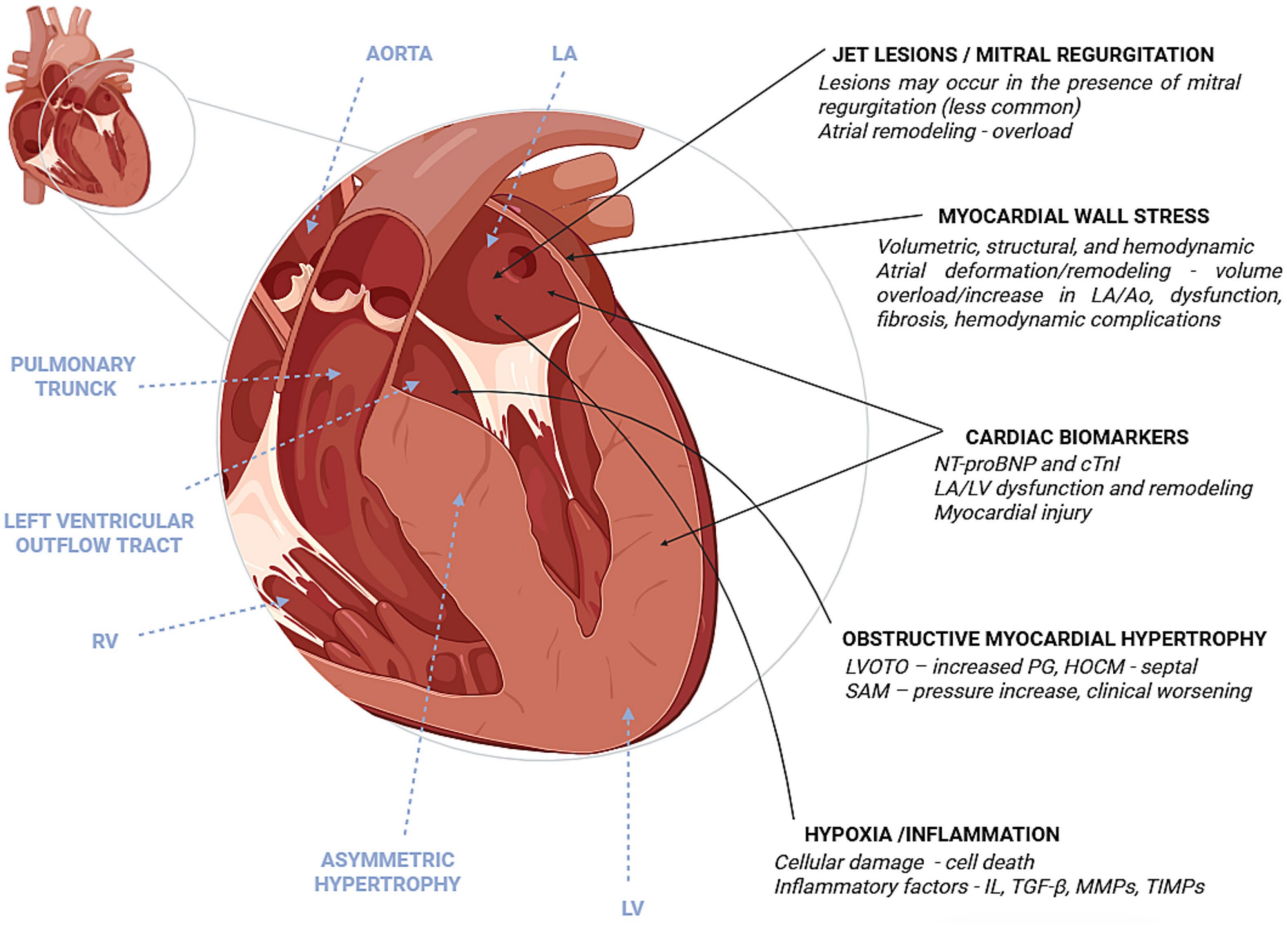
Schematic representation of the multifactorial causes of endothelial injury: regurgitant jet lesions (less common), wall stress/remodeling, left ventricular outflow obstruction, systolic anterior motion, hypoxia, and inflammation. RV, right ventricle; LA, left atrium; LV, left ventricle; LA/Ao, left atrium/aorta ratio; NT-proBNP, N-terminal pro-brain natriuretic peptide; cTnI, troponin I; PG, pressure gradient; HOCM, obstructive cardiomyopathy; IL, interleukin; TGF-β, transforming growth factor beta; MMPs, matrix metalloproteinases; TIMPs, tissue inhibitors of metalloproteinases. Created with BioRender.

Atrial endothelial injury, particularly on the left side, occurs in cats with HCM phenotype and arises from multiple mechanisms, including wall stress, hypoxia, inflammation, and jet lesions from mitral regurgitation (less commonly) (Figure 3). Atrial wall stress results from remodeling due to combined volume and pressure overload. Importantly, this overload is not due to increased pulmonary or systemic blood flow, but from altered ventricular geometry and impaired diastolic function. According to De Raffele et al. (47), LA dysfunction is a frequent finding in both humans and cats with HCM. Under these conditions, alterations in ventricular relaxation patterns and increased LV filling pressures develop, leading to secondary atrial involvement (47). Consequently, both feline and human patients with HCM may exhibit atrial deformation and remodeling, a process that is reflected by increased cardiac troponin I (cTnI) concentrations (Figure 3).

#### 2.1.1 Atrial wall stress and hemodynamic dysfunction

Assessment of atrial function is important condition associated with hemodynamic impairment, particularly in advanced stages of HCM. Distinct phases of the cardiac cycle can be evaluated to assess LV filling, including the reservoir (LV systole), conduit (early diastole), and booster pump phases (late diastole) (48). After LV pressure decreases at the onset of a new cardiac cycle and the aortic valve closes, the mitral valve opens, allowing blood to flow from the LA into the LV (conduit phase) (48). During this phase, atrial pressure declines, blood is passively directed into the LV, and ventricular pressure begins to rise (48). During late diastole, the atrial booster pump phase occurs, characterized by active atrial contraction that completes LV filling (48). When atrial mechanical dysfunction or increased stiffness occurs, these coordinated phases become impaired, leading to reduced cardiac efficiency and altered ventricular filling. In HCM phenotype, these alterations are worsened by myocardial hypertrophy and a reduced LV internal diameter (Figure 3). Atrial size and function are considered important prognostic indicators in both humans and cats with HCM, often associated with adverse outcomes (49–51). Veterinary studies show that increased atrial size, impaired function, and reduced LA fractional shortening are linked to poorer clinical outcomes (3, 6, 20, 52). Additionally, LA remodeling secondary to volume overload may serve as a useful marker of diastolic burden (53).

Similarly, veterinary evidence highlights the importance of assessing atrial size and function, particularly through the LA/Ao ratio, which is typically increased in HCM phenotype (2, 3). Another approach uses speckle-tracking echocardiography to evaluate atrial function via peak atrial longitudinal strain (PALS) (54). The study by Kiatsilapanan and Surachetpong (54) found that PALS was significantly reduced in the HCM group, particularly in relation to longitudinal deformation, with the exception of the septal roof and lateral wall segments. Weak correlations were found between PALS and diastolic velocities (e.g., pulmonary venous flow and mitral annular velocities), as well as systolic indices (e.g., transmitral flow). In contrast, negative correlations were identified with interventricular septal and posterior wall thickness, both measured at end-diastole. Notably, no significant association was found between PALS and left atrial size. Speckle-tracking is a valuable tool for assessing LA deformation in cats, with HCM phenotype patients demonstrating reduced strain values and a correlation between PALS and atrial function indices. This evidence suggests that strain-derived parameters reflect atrial mechanical impairment more sensitively than structural remodeling. PALS is an emerging parameter, although further validation is still required. In humans, further studies are required to confirm the prognostic utility of PALS (55). Atrial dilation is a recognized marker of disease severity in HCM, with LA/Ao values >1.8 associated with the occurrence of CHF or ATE (20, 56). Consequently, cats in advanced disease stages (e.g., ACVIM stages C and D) are expected to exhibit marked atrial enlargement due to chronic mechanical dysfunction. This condition is a target for therapeutic strategies aimed at improving diastolic function in HCM.

Emerging evidence supports the value of LA strain assessment in cats with the HCM phenotype, particularly for detecting early changes in atrial mechanical function. In the study by Tosuwan et al. (57), cats with ATE showed reduced LA reservoir strain (LASr), reflecting diminished atrial expansion and impaired reservoir performance. These cats showed alterations in conduit and contractile function, suggesting a more global impairment of atrial mechanics. In addition, LA strain parameters correlated with both conventional echocardiographic measurements and speckle-tracking indices. Importantly, LASr values < 11% were associated with ATE, with a strong performance in discriminating thromboembolic risk (AUC 0.93) (57). This evidence suggests that LASr is a promising experimental parameter for the assessment of early atrial dysfunction, particularly those with ATE. Another useful parameter to assess LA mechanical dysfunction in cats with the HCM phenotype is LA stiffness (LASt), although it still requires further validation. Evidence suggests that LASt is increased in cats with HCM, alongside elevated LV filling pressures, reduced LASr, and impaired LA mechanical function (58). In addition, LASt showed correlations with conventional echocardiographic parameters, including atrial dimensions, LAA flow velocity, as well as continuous-wave and tissue Doppler variables (58). The authors highlighted that, although LASt has recently been described in HCM, it shows potential for distinguishing healthy from diseased animals by enabling the assessment of atrial stiffness and remodeling. The recent study by Jeong et al. (59) also highlighted the presence of alterations in atrial mechanics in cats with HCM. The authors observed that affected cats showed evident reductions in ejection fraction and LASr, indicating that the atrium is also affected by the presence and progression of HCM in cats.

Atrial dynamics may also be influenced by ventricular factors, particularly LVOTO and SAM (Figure 3). In cats, LVOTO may contribute further to atrial remodeling and is a recognized clinical marker, especially involving the basal inferoseptal region near the aortic outflow (2, 9, 10, 60). Evidence indicates that cats with SAM exhibit increased peak LVOT velocities, atrial dilation, and impaired atrial function (12). LVOT velocity is dependent on the pressure gradient; thus, in the presence of SAM, intraventricular pressure and myocardial wall stress may increase (11). Depending on the degree of this pressure increase, secondary atrial effects may occur, potentially compromising atrial functional dynamics (11).

From a translational perspective, evidence supports the role of LVOTO and SAM in human atrial dysfunction (61, 62). In children with the HCM phenotype, Mazurkiewicz et al. (61) observed that the presence of LVOTO was associated with greater LA enlargement, increased myocardial wall thickness, and fibrosis. These patients also exhibited impaired atrial function, particularly reduced reservoir and conduit emptying fractions. Conversely, contractility and deformation mechanics were increased in children with obstructive HCM (61). In humans with HCM, the use of LA strain allows, as in cats, a more detailed assessment of atrial function, enabling the analysis of progression and risk stratification (63). Although valuable, evidence in humans highlights that conventional echocardiography does not allow dynamic assessment of atrial mechanics, requiring the use of tools that enable increasingly early analyses, especially for the LA (64). The analysis of atrial dynamics is more commonly described in humans. The LASr “quantifies elastic expansion of the atrial wall during ventricular systole, directly mirroring atrial compliance and left ventricular diastolic function”, allowing atrial coupling and stiffness to be assessed (64).

Evidence highlights that LASr in humans is associated with hemodynamic parameters such as systolic function, global deformation, and LV filling pressures. Patients with reduced maximal oxygen consumption and those with atrial fibrillation may exhibit altered myocardial deformation during both the conduit and pump phases of cardiac function (65). In humans, some limitations are described in the measurement of LASr such as variability of equipment and analysis software, heart rhythm, difficulties related to patient type, and anatomical and extracardiac conditions (64). Humans with HCM may present reduced deformation in the reservoir and pump phases, being independent factors for atrial fibrillation (≤8% for booster pump and ≤18% for reservoir) (66). The study by Raman et al. (66) highlighted that the analysis of LA deformation in humans with HCM, associated with age group, were independent predictors of atrial fibrillation risk. The authors proposed that routine analysis of atrial deformation may eventually contribute to risk assessment and future anticoagulant decision-making. However, this approach remains investigational and requires validation (66). Other evidence highlights that the assessment of LA deformability provides information related to adverse events in humans with HCM. Tian et al. (67) reported that reduced global longitudinal left atrial strain is independently associated with a higher incidence of adverse cardiovascular outcomes, with booster-phase strain ≤8.9% serving as a significant prognostic indicator.

The evidence suggests that atrial function in cats with the HCM phenotype should be considered multifactorial. It results from the interaction between volumetric factors (e.g., volume overload and dysfunction), structural changes (e.g., ventricular hypertrophy and obstruction), and hemodynamic alterations (e.g., flow disturbances and obstructive phenomena such as LVOTO and SAM) (Figure 3). Although these factors alone do not directly determine thrombus formation, they create a permissive environment for blood stasis and endothelial dysfunction.

#### 2.1.2 Cellular alterations and myocardial remodeling

Myocardial wall stress is associated with disease severity, particularly in the HCM phenotype, and with the release of biomarkers such as cardiac troponin I (e.g., cTnI) (Figure 3). According to Kittleson and Côté (15), myocardial wall stress occurs in cats with this phenotype, primarily due to underlying pathophysiological mechanisms. However, evidence of myocardial wall stress remains limited and is typically inferred from indirect markers. The presence of LVOTO in obstructive HCM is associated with higher pressure gradients compared to healthy cats, suggesting a potential causal role in increasing myocardial workload, particularly when assessed using deformation analysis (9, 10). Additionally, cTnI levels, an established marker of myocardial injury, are elevated in patients with LVOTO, indicating that obstruction may exacerbate myocardial damage (68). Ventricular wall stress is not always explicitly described in cats, although disease progression suggests its presence, particularly in advanced stages. The coexistence of LVOTO, ventricular hypertrophy, and elevated myocardial injury (cTnI) and cardiac function (NT-proBNP) biomarkers, recognized clinical markers in cats with the HCM phenotype, supports increased ventricular wall tension (13, 52, 69, 70) (Figure 3).

In humans, myocardial wall stress has been more extensively characterized, particularly in the ventricular myocardium, which may represent an early site of structural alteration. With regard to the atrial wall, evidence suggests heterogeneous stress distribution, with certain regions experiencing greater mechanical load than others (71). Peak wall stress approaching 100% has been reported in the regions of the left pulmonary veins and the ridge of the LAA, whereas other atrial regions show values ranging from 42 to 89% (71). This variability exists both across different regions and individuals. Furthermore, electrophysiological impairment has been described, whereby areas subjected to high mechanical stress exhibit low voltage and electrical scarring (71). These findings suggest a link between wall stress, electrical remodeling, and fibrosis. Recent studies in humans have also associated regions of elevated time-averaged wall shear stress (TAWSS), an experimental flow-based marker, with atrial fibrosis and electrical scarring (72). Atrial wall stress in humans depends on local geometric factors such as wall thickness and curvature, and is not fully explained by the traditional application of Laplace’s law (73).

Human data further indicate that myocardial wall stress is closely related to structural and hemodynamic alterations (74). The diversity of HCM phenotypes results in heterogeneous wall stress distributions, supporting the use of indices such as end-diastolic wall stress that account for regional curvature (74). Pharmacological interventions, such as mavacamten, have shown potential in reducing wall stress in humans with non-obstructive HCM, along with improvements in cTnI and NT-proBNP concentrations (75).

Although incompletely characterized in cats, myocardial wall stress likely contributes to myocardial injury and remodeling in the HCM phenotype. The hemodynamic alterations inherent to this phenotype, combined with local and systemic effects, atrioventricular remodeling, and obstructive phenomena, are sufficient to promote myocardial injury. These lesions arise from sustained mechanical stress associated with both hemodynamic burden and myocardial structural reorganization, including hypertrophy.

Additionally, other mechanisms may contribute to endothelial injury. Among these, hypoxia and activation of local inflammatory pathways are particularly relevant, often resulting from impaired myocardial perfusion and atrial remodeling (Figure 3). Notably, inflammatory and hypoxic processes in cats may occur even without primary genetic alterations in cardiomyocytes (76). Histopathological evaluation of myocardial tissue from cats with HCM phenotype has revealed myofiber disarray, cardiomyocyte hypertrophy, increased collagen deposition, and inflammatory infiltrates (76). In some cases, involvement of the mid-myocardial and subepicardial layers, as well as mural hypertrophy of small arterioles, has been documented in cats (76). These findings are supported by Kitz et al. (77), who further showed the presence of interstitial fibrosis, cardiomyocyte degeneration, and immunohistochemical expression of macrophages and other activated inflammatory cells in affected cats. Myocardial disarray, fibrosis, and ventricular mass in HCM phenotype can also be assessed using advanced imaging modalities, primarily research-based, such as micro-computed tomography (micro-CT) (78). Agreement with histopathological findings was observed, suggesting that this approach may be especially valuable in post-mortem evaluations (78).

Additionally, evidence suggests that affected cats exhibit alterations in vascular density, particularly within the microvasculature, which appears reduced in affected individuals (79). Interstitial cells may display fibroblastic or vascular phenotypes, with many expressing CD34 mRNA, supporting their involvement in disease progression (79). Furthermore, pro-inflammatory and pro-fibrotic mediators are overexpressed in the myocardial tissue of cats with HCM phenotype compared to controls (77). The resulting fibrotic state may impair electrical coupling between cells and predispose to arrhythmogenesis (80).

The role of inflammatory and remodeling mediators in feline cardiac disease has also been investigated by Fonfara et al. (81). Cats with heart disease show increased expression of myocardial markers, particularly interleukins (e.g., IL-18), growth factors (e.g., transforming growth factor-β; TGF-β), matrix metalloproteinases (MMPs; e.g., MMP-2, −3, −9, and −13), and tissue inhibitors of metalloproteinases (TIMPs; e.g., TIMP-1 and -3). Although active inflammation was not directly associated with structural myocardial changes in advanced stages, MMPs and TIMPs were implicated in remodeling processes (81). The authors stated “a systemic inflammatory state appears to elicit an inflammatory phenotype in the myocardium, whereas in HCM phenotype, the myocardium mediates its own remodeling”. Despite the benefits, the assessment of myocardial and inflammatory components remains largely experimental and may present limitations for routine clinical application.

To date, direct endothelial biomarkers are not well characterized in cats with the HCM phenotype. The presence of interstitial fibrosis, inflammation, inflammatory mediators, and myocardial wall stress suggests that the atrial endothelium undergoes both functional and structural alterations in a progression-dependent manner. This altered environment may predispose to local platelet activation (82), creating conditions for thrombogenesis even before overt hemodynamic abnormalities become evident.

### 2.2 Pre-SEC stasis

**Figure 4:**
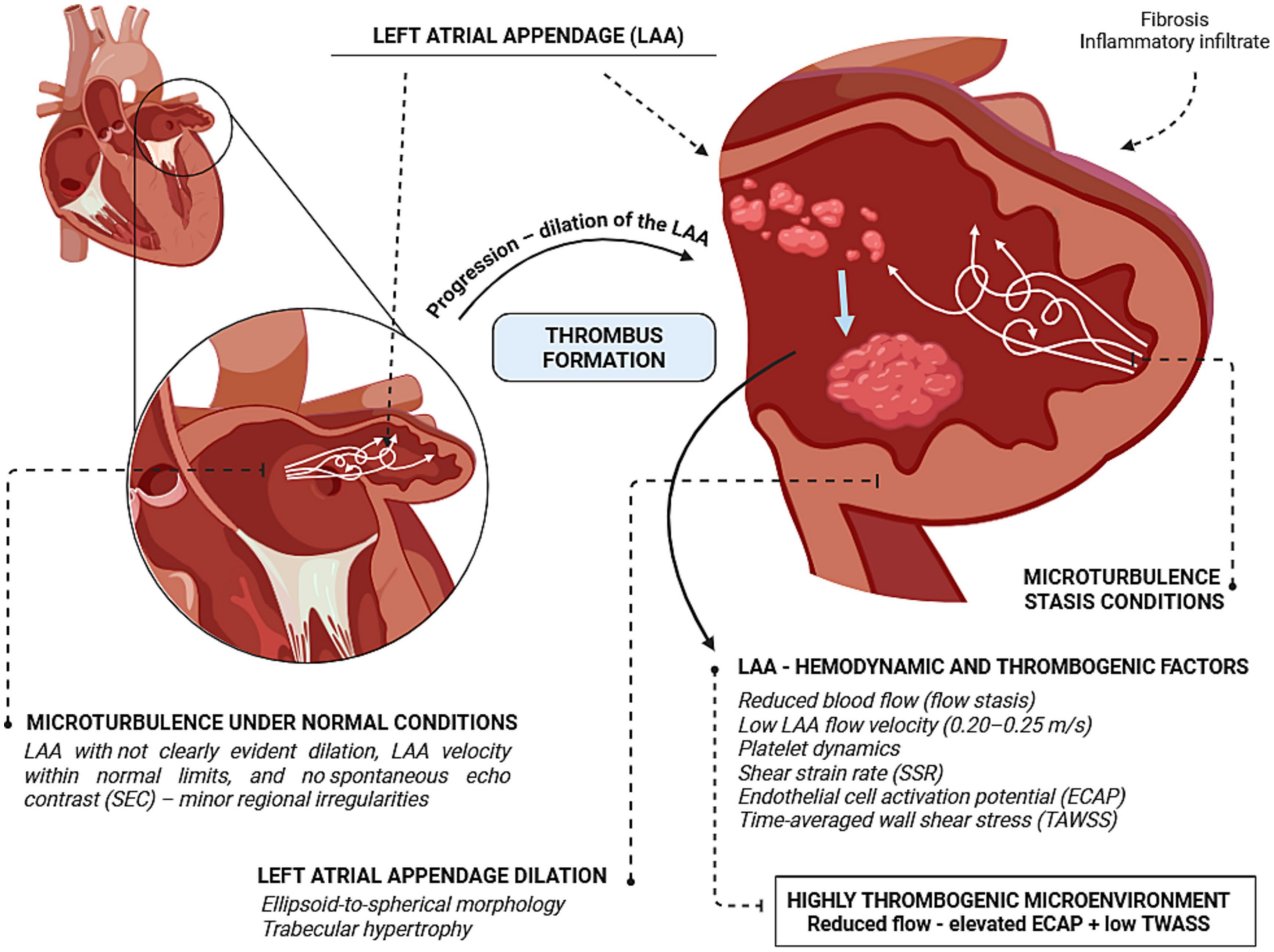
Schematic representation of manifestations associated with blood stasis, especially in the left atrial appendage. LAA, left atrial appendage; SEC, spontaneous echo contrast; SSR, shear strain rate; ECAP, endothelial cell activation potential; TAWSS, time-averaged wall shear stress. Created with BioRender.

Another key mechanism within Virchow’s triad is blood stasis, particularly within the LAA (Figure 2). According to Pons et al. (19), alterations in LA dynamics, such as those observed in patients with the HCM phenotype, can promote blood stasis, increasing the risk of thromboembolic events (Figure 4). However, the authors emphasize that the specific contributions of LAA structure and function to thrombus formation remain incompletely understood. The LAA exhibits considerable anatomical variability and has a central body with multiple lobes, in close anatomical relation to the pulmonary artery and the LV free wall (83). Ito and Suwa (84) further describe it as a “blind-ended, typically multi-lobed structure, connected anterolaterally to the left atrial (LA) body”.

In humans, the LAA has mechanical (contractile) and non-mechanical functions, including biomarker release and volume regulation, and should therefore be considered a functional organ rather than merely an embryological remnant (85). In cats, the LAA remains insufficiently studied, particularly because it is not routinely assessed in conventional echocardiographic examinations (86). Accordingly, Grassinger et al. (86) evaluated the LA and LAA in healthy and diseased cats, demonstrating that LAA volume increases with progressive dilation (healthy: 0.48 mL; severely dilated: 5.48 mL). According to their feline classification, normal LAA volume is ≤1 mL, mild dilation ranges from >1 to ≤2 mL, moderate dilation from >2 to ≤3 mL, and severe dilation >3 mL. Dilated LAA structures became more ellipsoid or spherical in shape, with primary involvement of the endocardium and myocardium, followed later by epicardial changes (86). Fibrosis and inflammatory infiltration were more pronounced in severely dilated appendages, and remodeling was more evident in the LAA than in the LA in cats. These morphological changes, including dilation and trabeculation, may enhance local stasis and create conditions favorable for thrombus formation.

According to Beigel et al. (87), effective LAA contraction under sinus rhythm and adequate flow velocity reduces the risk of thromboembolism by preventing blood stagnation. Conversely, human studies suggest that thrombogenesis is more likely in the presence of impaired contractility and reduced flow velocity (87). Assessment of LAA flow is recommended as a useful tool in evaluating thromboembolic risk (33). In humans, LAA flow velocity is influenced by multiple factors, including age, arrhythmias, and hemodynamic status (84).

In 2005, Schober and Maerz (83) were among the first to assess LAA flow velocity in cats, reporting a mean velocity of 0.47 ± 0.20 m/s in healthy individuals (range: 0.24–1.00 m/s). In a subsequent study, these authors observed that LAA flow patterns were more variable, often biphasic, and generally reduced in cats with cardiomyopathies (33). Cats with CHF exhibited lower LAA velocities, increased prevalence of SEC, and alterations in echocardiographic variables such as atrial size, fractional shortening, and indices of atrioventricular diastolic function. Atrial mechanical dysfunction progressed alongside disease severity and was associated with echocardiographic features linked to increased thromboembolic risk. Cats with SEC showed atrial mechanical dysfunction and peak LAA velocities ≤0.25 m/s (Figure 4). In some cases, velocities as low as 0.20 m/s were associated with advanced atrial mechanical dysfunction and were identified as an independent predictor of SEC within the LA (OR 30.1). Accordingly, an LAA velocity threshold of 0.25 m/s demonstrated 100% sensitivity and 69% specificity for SEC detection. These findings suggest that values between 0.20 and 0.25 m/s represent a clinically relevant range for assessing SEC risk in cats (Figure 4). Pharmacological interventions such as pimobendan may improve LAA velocity in cats, although further studies are warranted (88).

In humans, Lo Presti et al. (89) showed that following atrial or LAA wall injury associated with endothelial dysfunction, thrombus formation does not occur immediately because the threshold for platelet adhesion has not been reached. Once this initial phase is surpassed, thrombus formation begins and progresses rapidly. Human computational models indicate that thrombus development follows a dynamic pattern, including an initial growth phase, a transient deceleration, subsequent rapid expansion, potential obstruction of blood flow by thrombotic masses, and eventual stabilization (89). These models show that thrombus formation often originates at the distal tip of the LAA, highlighting its high thrombogenic potential due to localized stasis. However, these findings are currently derived from experimental computational simulations.

From a translational perspective, computational and translational human studies suggest that the presence of a thrombus may further alter local hemodynamics (shear strain rate – SSR) (89). As the thrombus enlarges, greater surface area interacts with blood flow, progressively increasing SSR, while the thrombus core exhibits solid-like behavior with local stasis (Figure 4). Over time, embolization may occur, with portions of the thrombus extending into the atrial cavity. Ultimately, thrombus stabilization occurs, occupying up to 45% of the computational domain and 65% of LAA volume (89). Further findings by Lo Presti et al. (89), indicate that thrombin concentrations vary within the LAA. Lower concentrations are observed near the atrial junction, whereas higher levels are found in intermediate and lateral regions, particularly during thrombus expansion. Other regions, such as the superior and inferior right portions, exhibit greater temporal variability in thrombin concentration. Similarly, in humans, activated platelet dynamics show heterogeneous distribution, with rapid accumulation in regions of early thrombus formation followed by a decline as platelets become incorporated into the thrombus. In contrast, regions with delayed or less pronounced thrombosis demonstrate slower or lower levels of platelet activation (89).

In cats, both the morphology and function of the LAA remain underexplored (19, 89). Pons et al. (19) identified marked differences between healthy cats and those with cardiomyopathy, including variations in cardiac weight, atrial and LAA volumes, and hemodynamic parameters at the ostium. In cats with cardiomyopathy, particularly symptomatic individuals, the LAA exhibited ostial dilation and reduced blood flow velocity, indicating conditions favorable for thrombogenesis (Figure 4). Cats with thrombi had lower LAA velocities than healthy individuals and intermediate values compared to CHF patients without thrombi. The same study (19) also evaluated the endothelial cell activation potential (ECAP) in cats, an experimental, computationally derived parameter reflecting endothelial susceptibility to thrombogenic activation. The distal LAA, particularly in trabeculated regions, demonstrated the highest ECAP values (Figure 4). In cats with the HCM phenotype, ECAP ranged from 0.4 to 1 Pa^−1^, indicating variability in endothelial activation. Higher flow velocities were observed within the LA, whereas lower velocities were present in the trabeculated regions of the LAA, corresponding inversely with time-averaged wall shear stress (TAWSS). Higher TAWSS values were concentrated in regions of faster flow, whereas thrombotic cases exhibited notably lower TAWSS within the LA, a pattern favoring thrombogenesis (Figure 4). Thus, cats with cardiomyopathy, particularly those symptomatic or with thrombi, displayed lower shear rates compared to controls (19).

Notably, even in the absence of overt LAA dilation, preserved flow velocity, or visible SEC, small regions of flow irregularity or microturbulence may still occur. These localized flow disturbances may create thrombogenic microenvironments before detectable SEC develops (Figure 4). Advanced Doppler techniques or computational modeling may help identify such subclinical alterations, enabling earlier detection of cats at increased thrombotic risk, although these approaches currently remain limited to advanced or investigational settings (19, 33, 83, 89).

Collectively, the findings reported by Pons et al. (19) provide preliminary insights into mechanisms that may render the LAA a site predisposed to thrombogenesis. The combination of high ECAP and low TAWSS in regions of reduced flow may define a highly thrombogenic microenvironment. Such conditions are commonly observed in cardiovascular disease due to altered hemodynamics, where reduced blood flow promotes thrombus formation. As in humans, the distal LAA in cats appears to be particularly susceptible to thrombogenesis due to local stasis and complex morphology.

### 2.3 Systemic hypercoagulability in cats with HCM phenotype

**Figure 5:**
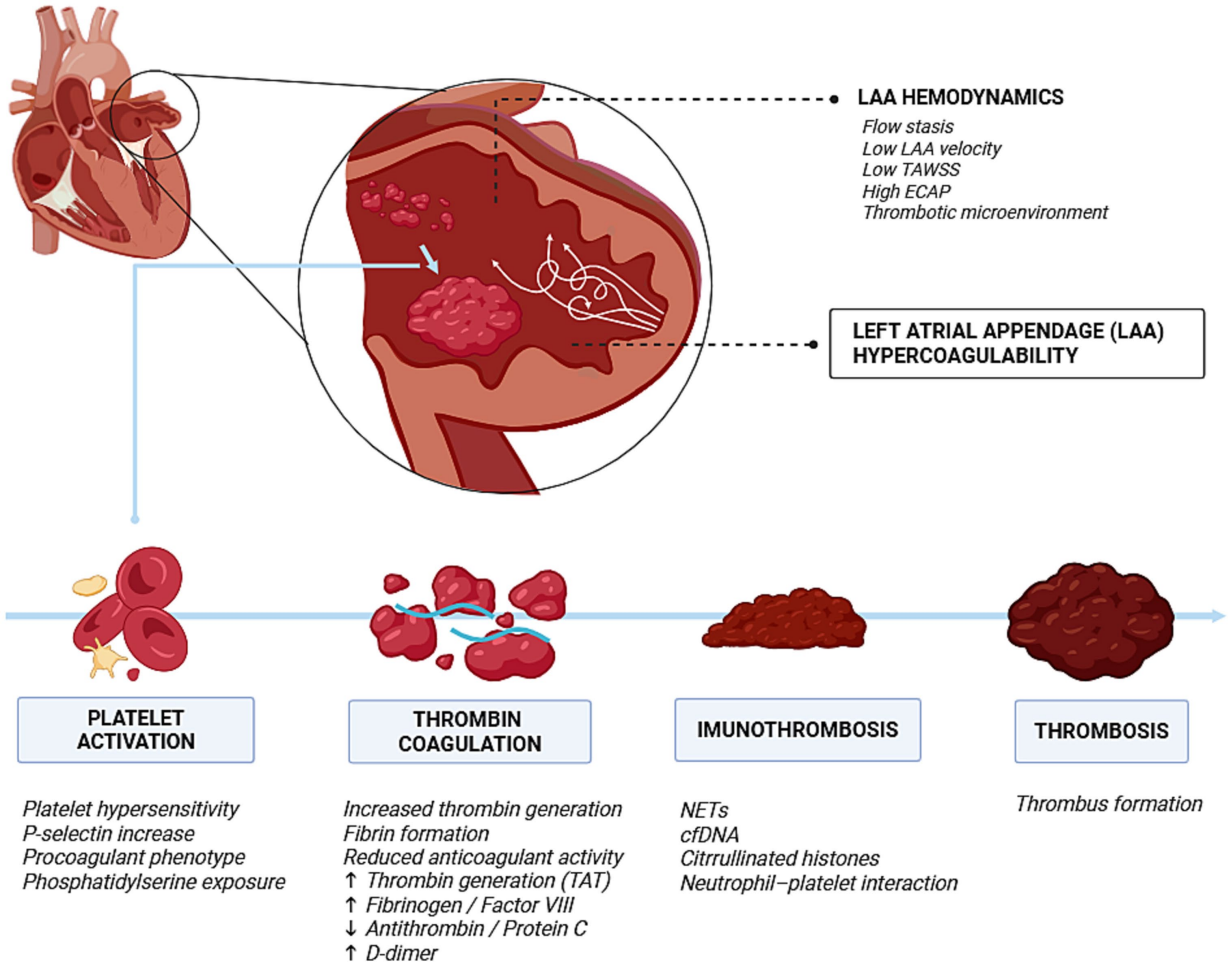
Schematic representation of processes and mechanisms associated with hypercoagulability, such as immunothrombosis, platelet heterogeneity, and procoagulant platelets. LAA, left atrial appendage; ECAP, endothelial cell activation potential; TAWSS, time-averaged wall shear stress; NETs, neutrophil extracellular traps; cfDNA, circulating cell-free DNA; TAT, thrombin–antithrombin complex. Created with BioRender.

The third pillar of Virchow’s triad is hypercoagulability, which represents an independent mechanism involved in thrombogenesis, and is thought to involve an imbalance between thrombin generation and anticoagulant pathways (29). Recent reviews have expanded the understanding of thrombogenesis in cats with HCM phenotype. These studies support the roles of immunothrombosis, platelet heterogeneity, and procoagulant platelets, as well as the development of novel biomarkers and targeted therapeutic approaches (25, 29) (Figure 5).

The imbalance between prothrombotic and antithrombotic factors underlies the hypercoagulable state and involves the interaction of multiple cellular and molecular components, including platelets, erythrocytes, and plasma proteins (24, 90). Hypercoagulability does not depend on ATE and instead represents a mechanistic substrate for thrombogenesis (90). According to Gavazza et al. (91), “the clinical incidence of thrombosis in cats has been connected with increased platelet hypersensitivity, decreased protein C and antithrombin activities, and increases in fibrinogen and factor VIII activity”.

Coagulation is highly dependent on platelet function. Following vascular injury, platelets adhere, become activated, and recruit additional platelets to the site of injury (24, 25, 38) (Figure 5). Platelet function in cats with HCM phenotype has been evaluated in previous studies. Jandrey et al. (92), using closure time measurements, reported higher values in affected cats compared to healthy controls, although without statistical significance. In contrast, Tablin et al. (82) demonstrated increased expression of P-selectin, CD41 + microvesicles, and soluble platelet–endothelial adhesion molecule-1, indicating enhanced platelet activation. P-selectin, in particular, is associated with fibrin stabilization and thrombus integrity (25). Nevertheless, the precise role of platelets in HCM phenotype and the clinical utility of these findings remains poorly understood.

Current evidence suggests that cats with HCM exhibit a procoagulant platelet phenotype, predisposing to thrombosis. Furthermore, platelet activation appears to correlate with disease severity (24). Although platelet-derived microparticles are associated with thrombin generation and thrombosis, Cremer et al. (93) did not identify significant differences between healthy and diseased cats. A recent review highlighted procoagulant platelets as potential therapeutic targets in companion animals. This finding underscores their relevance in thrombogenesis, particularly in conditions such as the HCM phenotype (25). Emerging evidence suggests that parameters such as PLCC and PLCR play an important role in disease progression and thromboembolic complications. A recent study highlighted that PLCR indices progressively increase with disease severity, in both clinical and subclinical disease (45). Regarding PLCC, evidence suggests that values are lower in patients with thromboembolic disease. The study also demonstrated a negative correlation between PLCR and platelet count, and a positive correlation between PLCC and platelet count. In addition, PLCC correlated with platelet distribution width and white blood cell count, suggesting a possible relationship between platelet activation and inflammation. PLCC evaluation demonstrated high diagnostic accuracy (AUC 0.8; cutoff ≤74 × 10^9^/L; sensitivity 71%; specificity 84%), in contrast to PLCR (AUC 0.56; cutoff >69%; sensitivity 85%; specificity 40%), which may influence their clinical applicability. The study further suggests that clinical manifestations and platelet activation may be preceded by an increased presence of large platelets, which are associated with thrombotic potential. However, these markers remain investigational and require further validation before routine clinical application.

Platelet stimulation with thrombin in combination with convulxin (a C-type lectin) induces a prothrombotic phenotype. Is characterized by mitochondrial membrane depolarization, increased P-selectin expression, and enhanced phosphatidylserine exposure (94). These findings suggest that, in cats with the HCM phenotype, platelets have a greater procoagulant activity upon activation. This finding supports the hypothesis that platelet activation may contribute to thrombus formation in conjunction with blood stasis and endothelial dysfunction. However, these mechanisms remain incompletely elucidated in cats.

In addition to platelet activation, inflammatory mechanisms have also been implicated in thrombogenesis, particularly through the formation of NETs in cats with HCM phenotype (Figure 5). Although NETs are part of the innate immune response, they play a critical role in thrombotic processes due to their ability to promote both inflammation and coagulation (95). Evidence suggests that NETs contribute to thrombus formation and propagation (95). Circulating cell-free DNA (cfDNA) and citrullinated histones, key components of NETs, may be present prior to overt thrombotic events. This evidence indicates their potential utility as investigational predictive biomarkers for ATE (95). In their study, Li et al. (95) demonstrated that cats with ATE exhibit significantly higher cfDNA concentrations compared to both HCM and healthy cats, with the highest levels observed in HCM patients with concurrent CHF. Approximately 75–83% of ATE cases showed cfDNA fragments ranging from 100 to 2000 base pairs. Histone concentrations were lower in HCM phenotype cats compared to those with ATE. Additionally, cfDNA levels were negatively correlated with LAA velocity and LA fractional shortening, and positively correlated with the LA/Ao ratio and to a lesser extent, neutrophil counts. These findings highlight the involvement of immunothrombotic pathways in the pathophysiology of thrombosis in cats with HCM phenotype and ATE (Figure 5).

Immunothrombosis has been proposed as a contributing mechanism of thrombogenesis, in which activation of the immune system, while attempting to contain potential pathogens, simultaneously promotes thrombus formation (24). This mechanism may contribute to microvascular thrombosis under conditions of dysregulated or exaggerated immune responses (Figure 5). Interactions between neutrophils and platelets within this framework may also be reflected in hematological indices associated with prognosis in cats with HCM and ATE. Yoon and Li (96) evaluated the NLR and platelet-to-neutrophil ratio (PNR) in healthy cats and those with HCM phenotype or ATE. They found that NLR values were highest in ATE, followed by HCM and healthy cats. The PNR was lower in ATE and showed no significant difference between affected and healthy groups. Cats with PNR values <40 had shorter survival times and a significantly increased risk of cardiac-related mortality. These findings suggest that PNR may be a more sensitive prognostic marker than NLR (96).

Secondary hemostasis also contributes to hypercoagulability in cats with HCM phenotype, particularly through increased thrombin generation (Figure 5). In this context, coagulation biomarkers have been investigated. Bédard et al. (97) demonstrated that the thrombin–antithrombin (TAT) complex is a sensitive marker of *in vivo* thrombin generation and hypercoagulability. They also reported the utility of D-dimer and fibrin degradation products, although laboratory availability may limit TAT measurement (97). Notably, atrial dimensions were not associated with hypercoagulability, and affected cats exhibited reduced antithrombin activity, supporting a prothrombotic state. Similarly, Stokol et al. (90) showed that cats with ATE exhibit evidence of both hypercoagulability and endothelial injury, particularly in the presence of SEC. Hypercoagulability was defined by the presence of at least two abnormal parameters (fibrinogen, factor VIII activity, TAT, and D-dimer) (Figure 5). Elevated levels of TAT, D-dimer, fibrinogen, factor VIII, and von Willebrand factor antigen (vWF:Ag) were observed, with TAT identified as the most sensitive indicator (90) (Figure 5). Atrial size was not a reliable indicator of hypercoagulability, with abnormalities detectable even in the absence of CHF or ATE. Although these biomarkers are promising, their routine clinical application in feline cardiology remains limited.

More recent experimental approaches, including the assessment of fibrin clot properties and thrombin generation, have been proposed to provide a more comprehensive characterization of the prothrombotic state. However, there remains a lack of studies evaluating these parameters in cats with HCM, representing a significant gap in current knowledge. In humans, Rubiś et al. (98) demonstrated that patients with HCM phenotype exhibit altered fibrin clot permeability, with the formation of more compact clots. Further studies are needed to clarify the relationship between coagulation abnormalities and thrombotic outcomes, especially in cats.

Although multiple experimental studies have investigated cellular and molecular markers of hypercoagulability in cats with the HCM phenotype, a more comprehensive functional assessment of coagulation dynamics remains poorly understood. Viscoelastic hemostatic assays, such as thromboelastography (TEG) and rotational thromboelastometry (ROTEM), provide a global evaluation of clot development and strength, providing insight into feline thrombogenesis. TEG allows continuous evaluation and recording of modifications in viscoelastic properties, as it incorporates the interaction between blood cells and plasma proteins (99). During TEG assessment, characteristics related to thrombin formation and kinetics, clot development, and maximum clot strength are evaluated, allowing a global analysis of the coagulation process (99). In general, cats with thrombogenic conditions may exhibit a hypercoagulable state, and viscoelastic analysis has been proposed as a useful tool for the evaluation of hypercoagulability and fibrinogenesis (82, 100). Nevertheless, there remains a gap in the viscoelastic evaluation of cats with HCM phenotype. These techniques currently remain primarily research tools in feline medicine.

In the case of thromboelastometry or ROTEM, viscoelastic properties are analyzed under conditions of reduced shear stress and represents an adaptation of TEG (99). It also allows the assessment of hyperfibrinolysis, described as an overactivation of the fibrinolytic system or a reduction in its physiological inhibitors (101). The use of the ROTEM delta analyzer has demonstrated reproducibility and may be applied for hemostatic assessment in cats, although minimal intra-observer variability should be considered (102). A Brazilian study evaluated the use of ROTEM in Maine Coon cats with HCM carrying or lacking the MYBPC3-A31P mutation and no evidence of hypercoagulability was identified (103). Therefore, the use of ROTEM in the analysis of cats with HCM requires further investigation before clinical application. Point-of-care viscoelastic tests may also be used because of their greater accessibility and lower cost; however, they may not correlate with TEG findings (100).

Methodological factors may also influence viscoelastic findings in cats. Previous studies demonstrated that kaolin-activated TEG may exhibit intra-assay variability and that sedation protocols may influence specific parameters (104). However, clinically relevant differences are not consistently observed in healthy cats (104). In addition, prolonged sample storage times may artificially promote hypercoagulable profiles. This evidence emphasizes the importance of standardized protocols and appropriate reference intervals in feline viscoelastic testing (105).

Global hemostatic assays, such as thrombin generation tests and viscoelastic methods, allow integrated evaluation of cellular and plasma components of coagulation. However, their utility may be limited by sample volume, timing constraints and practical application in cats. Although cats with HCM tend to be hypercoagulable, recent global hemostatic assays have revealed findings suggestive of hypocoagulability, particularly with concurrent ATE. Cats with ATE exhibited a hypocoagulable state, contrasting with the classical concept of Virchow’s triad (106). The authors observed that variables related to clot formation and maximum amplitude at 10 and 20 min were lower in cats with ATE, consistent with a hypocoagulable state. However, hypocoagulability may be affected by pre-analytical factors (e.g., operator error, sample collection, and processing), as well as by consumptive processes or metabolic alterations. These findings highlight the need for further investigation into the viscoelastic coagulation profile in cats with ATE.

Hypocoagulability in cats with cardiomyopathy and ATE is characterized by prolonged reaction times and altered kinetics in TEG, as well as changes in thrombin generation (107). A recent study by Langhorn et al. (107) demonstrated that cats with HCM and ATE exhibit a more pronounced hypocoagulable state. The authors emphasized that “hemostasis is a dynamic, complex, and balanced process with prothrombotic, fibrinolytic, and regulatory mechanisms constantly in play”, highlighting the multifactorial and dynamic nature of hemostasis. Although the presence of thrombin-antithrombin complexes is associated with coagulation activation and may indicate hypercoagulability, the study did not confirm a systemic hypercoagulable state. In addition, the authors suggested that the observed hypocoagulability is likely influenced by factors beyond platelet count and platelet activity alone, including alterations in coagulation factors. In this context, changes were identified in parameters related to reaction time and clot formation kinetics (107). Overall, the authors suggested that the hypocoagulable state observed may be associated with mechanisms such as platelet consumption/exhaustion and localized endocardial hypercoagulability. This may potentially interfere with the results of global hemostatic testing. These findings underscore the need for further investigation into viscoelastic profiles in feline patients.

From a translational perspective, in human medicine, TEG and ROTEM are applied across several clinical settings (e.g., surgical procedures); however, the data are not fully interchangeable (108). Evidence regarding TEG and ROTEM in patients with HCM remains scarce. Their clinical application has expanded particularly in situations involving thrombotic and bleeding risk assessment, especially in conditions in which prothrombin time provides limited information on coagulation, making them an alternative to conventional coagulation tests (108, 109). In peripheral arterial diseases, the use of TEG with platelet mapping may be useful in the adjustment of antiplatelet therapies, in addition to predicting outcomes in patients (110, 111). It is also important to highlight that many patients undergoing anticoagulant therapy may remain at risk for thrombosis due to the absence of adequate platelet function assessment (110). Additional applications include the evaluation of platelet function and thrombotic risk in carotid artery disease and stent placement, as well as the prediction of hemorrhagic risk and transfusion requirements (111). Other evidence highlight the potential benefits of TEG or ROTEM use in humans such as COVID-19, non-cardiac surgeries and use of blood products (112–115).

Overall, current evidence suggests that hypercoagulability in cats, with or without ATE, involves multiple mechanisms, including platelet activation, enhanced thrombin generation, and inflammatory pathways (Figure 5). However, recent findings also suggest that a hypocoagulable state may occur during certain stages, potentially due to platelet consumption or analytical limitations. These findings highlight the complexity of hemostatic alterations in cats with HCM phenotype.

## 3 Spontaneous echo contrast: marker or mechanism?

SEC represents a visible manifestation of blood stasis (14), arising from anatomical, hemodynamic, and biological factors (116) (Figure 1). SEC is a characteristic echocardiographic finding of abnormality mediated by blood stasis, reflecting a prothrombotic state (117, 118). Thrombotic risk correlates with SEC severity particularly within the LAA (118), due to its close relationship with blood flow dynamics (35) (Figure 3). SEC typically appears as “spontaneous, cloud-like echogenic swirling,” resulting from erythrocyte aggregation (118). In humans, it is frequently associated with atrial fibrillation due to altered atrial mechanics (118). Evidence also indicates that SEC within the LAA is associated with an increased risk of ischemic stroke in humans (119) (Figure 1).

In clinical practice, particularly in human medicine, SEC can be graded using semiquantitative echogenicity scoring systems. One widely used classification, proposed by Fatkin et al. (37), grades SEC from 0 to 4+: grade 0 (no echogenicity), 1 + (mild/minimal echogenicity – localized to the LAA or diffusely in the LA, often transient), 2 + (mild-to-moderate echogenicity – greater density than 1+), 3 + (moderate echogenicity – visible throughout the cardiac cycle, denser in the LAA than in the LA), and 4 + (severe echogenicity – intense density with markedly reduced LAA flow). Subsequently, using backscatter techniques, Klein et al. (120) quantified atrial SEC in humans. The study demonstrated progressively increased integrated backscatter values in accordance with severity, previously described qualitatively as mild, moderate, and severe.

More recently, emerging technologies in humans such as convolutional neural networks have demonstrated moderate performance in the detection of SEC; however, clinical application remains limited (117). Despite advances, the semiquantitative scoring system proposed by Fatkin et al. (37) remains the most widely used method in clinical practice due to its simplicity, reproducibility, and applicability. In feline medicine, however, SEC assessment is not standardized and is typically evaluated qualitatively (presence vs. absence), resulting in subjective, non-standardized interpretation without formal grading.

Ito and Suwa (116) emphasize that SEC helps guide management decisions in humans with cardiovascular disorders. This concept has been extrapolated to cats with cardiovascular diseases, particularly HCM phenotype and ATE (Figure 1). SEC has also been associated with inflammatory markers, although this association has been less extensively studied than those with hypercoagulability and blood stasis (116). Another relevant concept is left atrial sludge, described as an intermediate stage between SEC and overt thrombus formation, which may respond to anticoagulant therapy (34). While SEC reflects erythrocyte aggregation under low-flow conditions, left atrial sludge represents a subsequent stage characterized by fibrin formation and progression toward thrombosis (34).

Multiple studies have reported SEC in cases of ATE. Peck et al. (30) demonstrated that SEC in cats is linked to increased left ventricular internal diameter in systole, as well as decreased interventricular septal thickness and reduced fractional shortening. Additionally, univariate analysis indicated that SEC is associated with an increased risk of mortality in cats with acquired cardiomyopathies. Measurements such as left atrial fractional shortening and fractional area may predict thrombus formation. However, cutoff values in asymptomatic cats remain undefined prior to SEC development (31). Pharmacological management in cats, particularly combination therapy (e.g., rivaroxaban and clopidogrel) (28), reduces recurrence rates, decreases adverse events, and improves survival, especially in patients with multiple risk factors (≥ 2) (121). Clopidogrel, a P2Y12 inhibitor (adenosine diphosphate receptor inhibitor), and rivaroxaban, a factor Xa inhibitor of the coagulation cascade, have been recommended for use in cats with the HCM phenotype (2, 122, 123). Depending on disease severity, clopidogrel alone may not be sufficient for antithrombotic therapy, requiring combination with factor Xa inhibitors such as rivaroxaban (121). Evidence has shown that co-therapy with clopidogrel and rivaroxaban may promote clinical benefit with few adverse effects and increased survival time (121). Drugs such as aspirin and clopidogrel may decrease platelet function in healthy cats, with the effect being more evident for clopidogrel (124). Assessment of platelet function using PFA-100 and Plateletworks may be useful in cats receiving clopidogrel (124). Evidence suggests that the combined use of clopidogrel and rivaroxaban promotes a synergistic effect on thrombin generation and platelet activation (125). Thus, co-therapy with distinct mechanisms of action allows reduction of thrombin generation and blockade of platelet receptors, resulting in lower platelet aggregation and thrombotic potential (125). Furthermore, reduced platelet activation and thrombin generation likely contribute to improved atrial flow dynamics, reduced blood stasis, and a lower likelihood of SEC formation.

The development of SEC is best interpreted as a marker of blood stasis and low-flow conditions rather than a primary cause of thrombogenesis. SEC is part of the pathophysiological continuum of thrombogenesis but does not itself initiate thrombus formation. Its presence reflects advanced hemodynamic and structural alterations associated with thrombogenic risk. From a mechanistic standpoint, SEC results from erythrocyte aggregation secondary to reduced shear stress and increased fibrinogen concentration, making SEC visible in regions of sluggish flow.

Therefore, the presence of SEC should be considered a clinically relevant finding, supporting early intervention to reduce progression to ATE. However, it is important to recognize that SEC does not possess absolute predictive value, as its absence does not exclude thrombotic risk. Although clinically relevant, SEC absence does not exclude thrombotic risk. This progression advances to detectable stasis and may progress to thrombus formation.

## 4 Why does clinical practice still rely on smoke?

In clinical practice, the effects of Virchow’s triad associated with ATE in cats with the HCM phenotype is often inferred from the presence of SEC (Figure 1). Several factors help explain why the presence of SEC is still regarded as a key marker for thromboembolic risk in these patients. A major limitation is the lack of consistent coagulation, platelet, and inflammatory biomarkers capable of identifying the pre-thrombotic state at an early stage. Furthermore, much of the available evidence is derived from patients in advanced stages of disease or already classified as high-risk. This limits understanding of the early events involved in thrombogenesis. Another limitation of hemostatic testing in cats, including analytical variability and lack of standardization. These limitations limit routine clinical use. The assessment of thrombotic status in cats with HCM phenotype and/or ATE is more easily performed using echocardiography with Doppler, as it is less invasive and more readily applicable in clinical settings. Consequently, the identification of SEC remains a practical and accessible tool for determining thromboembolic risk, although it remains a relatively late marker in the disease process.

## 5 Clinical implications and future hypothesis

The role of endothelial dysfunction should be considered in the context of active thrombogenesis in affected cats. In clinical practice, thrombotic risk is usually recognized only after SEC becomes evident, a stage associated with an increased likelihood of thrombus formation. At this stage, SEC often prompts antithrombotic therapy, such as clopidogrel. Clopidogrel is recommended for cats considered at risk of ATE, including those with “moderate to severe LA enlargement, low LA FS%, low LA appendage velocities, SEC” (2). However, the consensus panel classified this recommendation as based on moderate-level agreement, reflecting limited evidence (2). Moreover, a prothrombotic state may persist in cats with the HCM phenotype even after the management of thromboembolic events, suggesting an underlying intrinsic susceptibility (Figure 2). In this context, improved risk stratification could help by improving clinical awareness and surveillance. It would also aid in the identification of patients who may be candidates for future prospective evaluation of earlier therapeutic strategies.

A potential pre-SEC thrombogenic stage remains hypothetical and requires prospective validation. Several parameters could improve risk stratification, including LA size, atrial function, LAA flow velocity, and the evaluation of biomarkers related to coagulation and atrial function/injury (Figures 2, 3). Additionally, experimental markers associated with fibrosis, myocardial remodeling, inflammation, and disease stage may improve characterization of thrombotic risk, potentially integrated into a scoring system. Despite this, studies integrating these parameters in cats with the HCM phenotype remain scarce, representing an important knowledge gap. Together, these findings support the hypothesis of a previously underrecognized “pre-SEC” thrombogenic stage in affected cats. However, the clinical applicability and therapeutic implications of this concept require prospective validation.

Current evidence regarding atrial remodeling, SEC, and ATE in cats, particularly in the context of Virchow’s Triad, remains incompletely understood. In addition, the temporal sequence of events involving atrial remodeling, changes in the prothrombotic state, detection of SEC, and occurrence of ATE remains unclear in cats. A considerable proportion of the available evidence is derived from human studies or experimental and computational models, while cat-specific data remain limited. Interpretation of the available studies is also complicated by the lack of standardization of feline cardiovascular and hemostatic biomarkers, as well as by species-specific physiological limitations. Although concepts of Virchow’s triad may be extrapolated from human medicine to cats, additional prospective and longitudinal studies are still required to improve clinical application of these findings.

## 6 Take-home message

Features of Virchow’s triad are present in cats with the HCM phenotype, with or without associated ATE. Its activation should be considered in patients exhibiting conditions associated with endothelial injury, enhanced coagulation, and alterations in blood flow. Thromboembolic risk stratification is more established in humans and is often associated with SEC; however, this finding represents a relatively late marker. Affected cats already exhibit hemodynamic and biological alterations that support the transition from a pre-thrombotic state to overt thrombogenesis. Prospective studies are still required to validate early thrombogenic markers and improve feline thromboembolic risk stratification. These approaches should incorporate coagulation, inflammatory, and platelet biomarkers, alongside clinical parameters such as LA size, function, and LAA flow velocity. These variables could be incorporated into a risk scoring system. Therefore, thrombogenesis in cats with HCM should be understood as a dynamic and multifactorial process.

## References

[1] de Sousa FG Muzzi RAL de Araújo RB Faleiros RR Queiroz FSF Beier SL. Linking clinical and imaging diagnostic assessments of the feline hypertrophic cardiomyopathy phenotype. Front Vet Sci. (2025) 12:1720886. doi: 10.3389/fvets.2025.1720886, 41487485 PMC12756110

[2] Luis Fuentes V Abbott J Chetboul V Côté E Fox PR Häggström J . ACVIM consensus statement guidelines for the classification, diagnosis, and management of cardiomyopathies in cats. J Vet Intern Med. (2020) 34:1062–77. doi: 10.1111/jvim.15745, 32243654 PMC7255676

[3] Novo Matos J Payne JR. Predicting development of hypertrophic cardiomyopathy and disease outcomes in cats. Vet Clin N Am - Small Anim Pract. (2023) 53:1277–92. doi: 10.1016/j.cvsm.2023.05.012, 37500329

[4] Payne JR Brodbelt DC Luis Fuentes V. Cardiomyopathy prevalence in 780 apparently healthy cats in rehoming centres (the CatScan study). J Vet Cardiol. (2015) 17:S244–57. doi: 10.1016/j.jvc.2015.03.008, 26776583

[5] Novo Matos J Payne JR Seo J Luis Fuentes V. Natural history of hypertrophic cardiomyopathy in cats from rehoming centers: the CatScan II study. J Vet Intern Med. (2022) 36:1900–12. doi: 10.1111/jvim.16576, 36315028 PMC9708425

[6] Payne JR Borgeat K Brodbelt DC Connolly DJ Luis Fuentes V. Risk factors associated with sudden death vs. congestive heart failure or arterial thromboembolism in cats with hypertrophic cardiomyopathy. J Vet Cardiol. (2015) 17:S318–28. doi: 10.1016/j.jvc.2015.09.008, 26776589

[7] Fox PR Keene BW Lamb K Schober KE Chetboul V Luis Fuentes V . Long-term incidence and risk of noncardiovascular and all-cause mortality in apparently healthy cats and cats with preclinical hypertrophic cardiomyopathy. J Vet Intern Med. (2019) 33:2572–86. doi: 10.1111/jvim.15609, 31605422 PMC6872868

[8] Fox PR Keene BW Lamb K Schober KA Chetboul V Luis Fuentes V . International collaborative study to assess cardiovascular risk and evaluate long-term health in cats with preclinical hypertrophic cardiomyopathy and apparently healthy cats: the REVEAL study. J Vet Intern Med. (2018) 32:930–43. doi: 10.1111/jvim.15122, 29660848 PMC5980443

[9] Hirose M Watanabe M Takeuchi A Yokoi A Terai K Matsuura K . Differences in the impact of left ventricular outflow tract obstruction on intraventricular pressure gradient in feline hypertrophic cardiomyopathy. Animals. (2024) 14:3320. doi: 10.3390/ani14223320, 39595372 PMC11591385

[10] Saito T Suzuki R Yuchi Y Fukuoka H Satomi S Teshima T . Comparative study of myocardial function in cases of feline hypertrophic cardiomyopathy with and without dynamic left-ventricular outflow-tract obstruction. Front Vet Sci. (2023) 10:1191211. doi: 10.3389/fvets.2023.1191211, 37426078 PMC10324663

[11] Ferasin L Kilkenny E Ferasin H. Evaluation of N-terminal prohormone of brain natriuretic peptide and cardiac troponin-I levels in cats with systolic anterior motion of the mitral valve in the absence of left ventricular hypertrophy. J Vet Cardiol. (2020) 30:23–31. doi: 10.1016/j.jvc.2020.05.001, 32645686

[12] Seo J Novo Matos J Munday JS Hunt H Connolly DJ Luis Fuentes V. Longitudinal assessment of systolic anterior motion of the mitral valve in cats with hypertrophic cardiomyopathy. J Vet Intern Med. (2024) 38:2982–93. doi: 10.1111/jvim.17203, 39325030 PMC11586536

[13] Seo J Payne JR Novo Matos J Fong WW Connolly DJ Luis Fuentes V. Biomarker changes with systolic anterior motion of the mitral valve in cats with hypertrophic cardiomyopathy. J Vet Intern Med. (2020) 34:1718–27. doi: 10.1111/jvim.15807, 32822105 PMC7517492

[14] Kittleson MD Côté E. The feline cardiomyopathies: 1. General concepts. J Feline Med Surg. (2021) 23:1009–27. doi: 10.1177/1098612X211021819, 34693806 PMC8723176

[15] Kittleson MD Côté E. The feline cardiomyopathies: 2. Hypertrophic cardiomyopathy. J Feline Med Surg. (2021) 23:1028–51. doi: 10.1177/1098612X211020162, 34693811 PMC8642168

[16] Gaia de Sousa F. Mendes A. C. R. Carvalho L. P. de Beier S. L. (2025). Clinical-diagnostic and therapeutic advances in feline hypertrophic cardiomyopathy. Vet Sci, 12:289. doi: 10.3390/vetsci12030289 40267000 PMC11946439

[17] Carter R Partington C Novo Matos J. Accuracy of combined signalment, physical examination and cardiac biomarkers in screening cats with asymptomatic murmurs for cardiac disease. J Vet Intern Med. (2026) 40:aalaf037. doi: 10.1093/jvimsj/aalaf037, 41742490

[18] Seo J Kurosawa TA Borgeat K Novo Matos J Hutchinson JC Arthurs OJ . Clinical signs associated with severe ST segment elevation in three cats with a hypertrophic cardiomyopathy phenotype. J Vet Cardiol. (2024) 54:30–7. doi: 10.1016/j.jvc.2024.05.003, 39004067

[19] Pons MI Olivares AL Mill J Matos JN Garcia-Canadilla P Cerrada I . Cardiomyopathy and thrombogenesis in cats through left atrial morphological and fluid dynamics analysis. Sci Rep. (2025) 15:12263. doi: 10.1038/s41598-025-96245-7, 40210661 PMC11986047

[20] Payne JR Borgeat K Connolly DJ Boswood A Dennis S Wagner T . Prognostic indicators in cats with hypertrophic cardiomyopathy. J Vet Intern Med. (2013) 27:1427–36. doi: 10.1111/jvim.12215, 24134821

[21] Guillaumin J. Feline aortic thromboembolism: recent advances and future prospects. J Feline Med Surg. (2024) 26:1–13. doi: 10.1177/1098612X241257878, 38857617 PMC11274361

[22] Kumar DR Hanlin ER Glurich I Mazza JJ Yale SH. Virchow’s contribution to the understanding of thrombosis and cellular biology. Clin Med Res. (2010) 8:168–72. doi: 10.3121/cmr.2009.866, 20739582 PMC3006583

[23] Zabixullaevich KR Dilshodovich KH Qizi XRM. Mechanism of the Virchow’s triad in the development of thrombosis. Int J Med Sci Clin Res. (2025) 5:173–8. doi: 10.37547/ijmscr/Volume05Issue11-32

[24] Shaverdian M Li RHL. Preventing cardiogenic thromboembolism in cats: literature gaps, rational recommendations, and future therapies. Vet Clin N Am - Small Anim Pract. (2023) 53:1309–23. doi: 10.1016/j.cvsm.2023.06.002, 37516545

[25] Shaverdian M Viall A Li RHL. Are procoagulant platelets an emerging therapeutic target? A general review with an emphasis on their clinical significance in companion animals. Int J Mol Sci. (2025) 26:8776. doi: 10.3390/ijms26188776, 41009344 PMC12470208

[26] Hogan DF. Feline cardiogenic arterial thromboembolism: prevention and therapy. Vet Clin North Am - Small Animal Pract. (2017) 47:1065–82. doi: 10.1016/j.cvsm.2017.05.001, 28662872

[27] Hogan DF Fox PR Jacob K Keene B Laste NJ Rosenthal S . Secondary prevention of cardiogenic arterial thromboembolism in the CAT: the double-blind, randomized, positive-controlled feline arterial thromboembolism; clopidogrel vs. aspirin trial (FAT CAT). J Vet Cardiol. (2015) 17:S306–17. doi: 10.1016/j.jvc.2015.10.004, 26776588

[28] Brainard BM Coleman AE Kurosawa A Rush JE Hogan DF Brooks MB . Therapy with clopidogrel or rivaroxaban has equivalent impacts on recurrence of thromboembolism and survival in cats following cardiogenic thromboembolism: the SUPERCAT study. J Am Vet Med Assoc. (2025) 263:1–10. doi: 10.2460/javma.24.09.0584, 39693732

[29] Yeh NS Shaverdian M Li RHL. Evolving FATE: a new lens on the pathogenesis and management of feline cardiogenic arterial thromboembolism. Animals. (2025) 15:1630. doi: 10.3390/ani15111630, 40509097 PMC12153538

[30] Peck CM Nielsen LK Quinn RL Laste NJ Price LL. Retrospective evaluation of the incidence and prognostic significance of spontaneous echocardiographic contrast in relation to cardiac disease and congestive heart failure in cats: 725 cases (2006-2011). J Vet Emerg Crit Care (San Antonio). (2016) 26:704–12. doi: 10.1111/vec.12509, 27479924

[31] Colakoglu E Sevim K Kaya U. Assessment of left atrial size, left atrial volume and left ventricular function, and its relation to spontaneous echocardiographic contrast in cats with hypertrophic cardiomyopathy: a preliminary study. Pol J Vet Sci. (2024) 27:487–90. doi: 10.24425/pjvs.2024.151743, 39736131

[32] Tan AWK Li RHL Ueda Y Stern JA Hussain M Haginoya S . Platelet priming and activation in naturally occurring thermal burn injuries and wildfire smoke exposure is associated with intracardiac thrombosis and spontaneous echocardiographic contrast in feline survivors. Front Vet Sci. (2022) 9:892377. doi: 10.3389/fvets.2022.892377, 35909698 PMC9329816

[33] Schober KE Maerz I. Assessment of left atrial appendage flow velocity and its relation to spontaneous echocardiographic contrast in 89 cats with myocardial disease. J Vet Intern Med. (2006) 20:120–30. doi: 10.1111/j.1939-1676.2006.tb02831.x, 16496931

[34] Melidoro P Lip GYH Montarello N Rajani R Klis M Williams SE . Left atrial spontaneous echo contrast: pathogenesis, detection, and modelling. Thromb Haemost. (2025). doi: 10.1055/a-2688-6741, (Online ahead of print). 40897348

[35] Watson T Shantsila E Lip GY. Mechanisms of thrombogenesis in atrial fibrillation: Virchow’s triad revisited. Lancet. (2009) 373:155–66. doi: 10.1016/S0140-6736(09)60040-4, 19135613

[36] Schulman S Makatsariya A Khizroeva J Bitsadze V Kapanadze D. The basic principles of pathophysiology of venous thrombosis. Int J Mol Sci. (2024) 25:11447. doi: 10.3390/ijms252111447, 39519000 PMC11547114

[37] Fatkin D Kelly RP Feneley MP. Relations between left atrial appendage blood flow velocity, spontaneous echocardiographic contrast and thromboembolic risk in vivo. JACC. (1994) 23:961–9. doi: 10.1016/0735-1097(94)90644-0, 8106703

[38] van der Meijden PEJ Heemskerk JWM. Platelet biology and functions: new concepts and clinical perspectives. Nat Rev Cardiol. (2019) 16:166–79. doi: 10.1038/s41569-018-0110-0, 30429532

[39] Taher AT Cappellini MD Bou-Fakhredin R Coriu D Musallam KM. Hypercoagulability and vascular disease. Hematol Oncol Clin North Am. (2018) 32:237–45. doi: 10.1016/j.hoc.2017.11.001, 29458729

[40] Zhou C Zhou Y Ma W Liu L Zhang W Li H . Revisiting Virchow’s triad: exploring the cellular and molecular alterations in cerebral venous congestion. Cell Biosci. (2024) 14:131. doi: 10.1186/s13578-024-01314-5, 39444013 PMC11515517

[41] Lurie JM Png CYM Subramaniam S Chen S Chapman E Aboubakr A . Virchow’s triad in “silent” deep vein thrombosis. J Vasc Surg Venous Lymphat Disord. (2019) 7:640–5. doi: 10.1016/j.jvsv.2019.02.011, 31078515

[42] Donadini MP Calcaterra F Romualdi E Ciceri R Cancellara A Lodigiani C . The link between venous and arterial thrombosis: is there a role for endothelial dysfunction? Cells. (2025) 14:144. doi: 10.3390/cells14020144, 39851572 PMC11763525

[43] Stone J Hangge P Albadawi H Wallace A Shamoun F Knuttien MG . Deep vein thrombosis: pathogenesis, diagnosis, and medical management. Cardiovasc Diagn Ther. (2017) 7:S276–84. doi: 10.21037/cdt.2017.09.01, 29399531 PMC5778510

[44] Borgeat K Wright J Garrod O Payne JR Fuentes VL. Arterial thromboembolism in 250 cats in general practice: 2004-2012. J Vet Intern Med. (2014) 28:102–8. doi: 10.1111/jvim.12249, 24237457 PMC4895537

[45] Algan D Varlik T Koch J Yilmaz Z. Diagnostic and prognostic significance of platelet large cell count and ratio in cats across stages of hypertrophic cardiomyopathy. Res Vet Sci. (2025) 197:105942. doi: 10.1016/j.rvsc.2025.105942

[46] Liu L Liu Z Chen X He S. Thromboembolism in patients with hypertrophic cardiomyopathy. Int J Med Sci. (2021) 18:727–35. doi: 10.7150/ijms.50167, 33437207 PMC7797548

[47] De Raffele M Teis A Cediel G Weerts J Conte C Juncà G . Left atrial remodelling and function in various left ventricular hypertrophic phenotypes. Eur Heart J Cardiovasc Imaging. (2025) 26:853–62. doi: 10.1093/ehjci/jeaf033 39874262

[48] Farhad H Seidelmann SB Vigneault D Abbasi SA Yang E Day SM . Left atrial structure and function in hypertrophic cardiomyopathy sarcomere mutation carriers with and without left ventricular hypertrophy. J Cardiovasc Magn Reson. (2017) 19:107. doi: 10.1186/s12968-017-0420-0, 29284499 PMC5745877

[49] Badran HM Soltan G Almeleigi R Faheem N Yacoub MH. Prognostic significance of left ventricular end diastolic pressure using E/E’ in patients with hypertrophic cardiomyopathy. Echocardiography. (2019) 36:2167–75. doi: 10.1111/echo.14539, 31742769

[50] Zhou D Yang W Yang Y Yin G Li S Zhuang B . Left atrial dysfunction may precede left atrial enlargement and abnormal left ventricular longitudinal function: a cardiac MR feature tracking study. BMC Cardiovasc Disord. (2022) 22:99. doi: 10.1186/s12872-022-02532-w, 35282817 PMC8919633

[51] Elkareem TSA Habib S Shehata A Elhady F. Detection of left atrial Remodeling by three-dimensional echocardiography in symptomatic patients known to had non-obstructive hypertrophic cardiomyopathy. Cardiol Res. (2024) 15:396–403. doi: 10.14740/cr1690, 39420978 PMC11483119

[52] Ironside V Tricklebank P Boswood A. Risk indictors in cats with preclinical hypertrophic cardiomyopathy: a prospective cohort study. J Feline Med Surg. (2021) 23:149–59. doi: 10.1177/1098612X20938651, 32696726 PMC10741343

[53] Sachdev V Shizukuda Y Brenneman CL Birdsall CW Waclawiw MA Arai AE . Left atrial volumetric remodeling is predictive of functional capacity in nonobstructive hypertrophic cardiomyopathy. Am Heart J. (2005) 149:730–6. doi: 10.1016/j.ahj.2004.07.017, 15990760

[54] Kiatsilapanan A Surachetpong SD. Assessment of left atrial function in feline hypertrophic cardiomyopathy by using two- dimensional speckle tracking echocardiography. BMC Vet Res. (2020) 16:344. doi: 10.1186/s12917-020-02557-3, 32948164 PMC7501631

[55] Essayagh B Resseguier N Michel N Casalta AC Renard S Donghi V . Left atrial dysfunction as marker of poor outcome in patients with hypertrophic cardiomyopathy. Arch Cardiovasc Dis. (2021) 114:96–104. doi: 10.1016/j.acvd.2020.06.004, 33039326

[56] Luis Fuentes V Wilkie LJ. Asymptomatic hypertrophic cardiomyopathy: diagnosis and therapy. Vet Clin N Am - Small Anim Pract. (2017) 47:1041–54. doi: 10.1016/j.cvsm.2017.05.002, 28662873

[57] Tosuwan J Surachetpong SD Hunprasit V. Assessment of left atrial myocardial deformation using two-dimensional speckle-tracking echocardiography in cats with cardiogenic and non-cardiogenic arterial thromboembolism. Int J Vet Sci Med. (2023) 11:11–22. doi: 10.1080/23144599.2023.2196853, 37025927 PMC10071954

[58] Tohthong P Tosuwan J Surachetpong SD. Left atrial stiffness as a novel echocardiographic parameter of atrial dysfunction in cats with hypertrophic cardiomyopathy: a two-dimensional speckle-tracking study. Vet World. (2025) 18:4046–55. doi: 10.14202/vetworld.2025.4046-4055, 41716159 PMC12913819

[59] Jeong CR Shin YJ Park C. Comparative study of speckle tracking echocardiography in Normal and hypertrophic cardiomyopathy cats. Vet Sci. (2026) 13:277. doi: 10.3390/vetsci13030277, 41893695 PMC13030113

[60] Stern JA Rivas VN Kaplan JL Ueda Y Oldach MS Ontiveros ES . Hypertrophic cardiomyopathy in purpose-bred cats with the A31P mutation in cardiac myosin binding protein-C. Sci Rep. (2023) 13:10319. doi: 10.1038/s41598-023-36932-5, 37365215 PMC10293195

[61] Mazurkiewicz Ł Ziółkowska L Petryka J Śpiewak M Małek Ł Kubik A . Biatrial performance in children with hypertrophic cardiomyopathy: CMR study. Eur Radiol. (2018) 28:5148–59. doi: 10.1007/s00330-018-5519-7, 29882072 PMC6223845

[62] Muresan I Agoston R Serban A Mot SCD Costea S Zlibut A . Evaluation and implications of mitral regurgitation using cMRI in patients with hypertrophic cardiomyopathy. Eur Rev Med Pharmacol Sci. (2023) 27:4006–18. doi: 10.26355/eurrev_202305_32306 37203824

[63] Jain V Ghosh R Gupta M Saijo Y Bansal A Farwati M . Contemporary narrative review on left atrial strain mechanics in echocardiography: cardiomyopathy, valvular heart disease and beyond. Cardiovasc Diagn Ther. (2021) 11:924–38. doi: 10.21037/cdt-20-461, 34295714 PMC8261755

[64] Sonaglioni A Nicolosi GL. Left atrial reservoir strain in cardiovascular and systemic disease: advances and clinical applications from physiology to practice. Rev Cardiovasc Med. (2025) 26:46198. doi: 10.31083/rcm46198, 41524057 PMC12781020

[65] Tayal B Malahfji M Buergler JM Shah DJ Nagueh SF. Hemodynamic determinants of left atrial strain in patients with hypertrophic cardiomyopathy: a combined echocardiography and CMR study. PLoS One. (2021) 16:e0245934. doi: 10.1371/journal.pone.0245934, 33566865 PMC7875429

[66] Raman B Smillie RW Mahmod M Chan K Ariga R Nikolaidou C . Incremental value of left atrial booster and reservoir strain in predicting atrial fibrillation in patients with hypertrophic cardiomyopathy: a cardiovascular magnetic resonance study. J Cardiovasc Magn Reson. (2021) 23:109. doi: 10.1186/s12968-021-00793-6, 34635131 PMC8504076

[67] Tian D Zhang JY He YF Xiong ZQ Zhao M Hu S . Predictive value of left atrial strain analysis in adverse clinical events in patients with hypertrophic cardiomyopathy: a CMR study. BMC Cardiovasc Disord. (2023) 23:42. doi: 10.1186/s12872-023-03069-2, 36690952 PMC9869521

[68] Satomi S Suzuki R Yuchi Y Yoshii Y Kanno H Teshima T . Relationship between cardiac troponin I concentration and myocardial function in hypertrophic cardiomyopathy cats with or without left ventricular outflow tract obstruction. Animals. (2025) 15:1313. doi: 10.3390/ani15091313, 40362128 PMC12071132

[69] Abdelhaleem SW Elsharkawy SH Salem SI Bashandy MM. Evaluation of some cardiac biomarkers in cats with primary and secondary cardiomyopathies. Vet J. (2025) 314:106441. doi: 10.1016/j.tvjl.2025.106441, 40983221

[70] Stack JP Fries RC Kruckman L Kadotani S Wallace G. Galectin-3 as a novel biomarker in cats with hypertrophic cardiomyopathy. J Vet Cardiol. (2023) 48:54–62. doi: 10.1016/j.jvc.2023.06.003, 37480722

[71] Hunter RJ Liu Y Lu Y Wang W Schilling RJ. Left atrial wall stress distribution and its relationship to electrophysiologic remodeling in persistent atrial fibrillation. Circ Arrhythm Electrophysiol. (2012) 5:351–60. doi: 10.1161/CIRCEP.111.965541, 22294615

[72] Adamopoulos D Rovas G Johner N Müller H Deux JF Crowe LA . Left atrial wall shear stress correlates with fibrosis in patients with atrial fibrillation. Nat Cardiovasc Res. (2025) 4:677–88. doi: 10.1038/s44161-025-00651-z, 40360793 PMC12170334

[73] Augustin CM Fastl TE Neic A Bellini C Whitaker J Rajani R . The impact of wall thickness and curvature on wall stress in patient-specific electromechanical models of the left atrium. Biomech Model Mechanobiol. (2020) 19:1015–34. doi: 10.1007/s10237-019-01268-5, 31802292 PMC7203597

[74] Zhao X Tan RS Tang HC Teo SK Su Y Wan M . Left ventricular wall stress is sensitive marker of hypertrophic cardiomyopathy with preserved ejection fraction. Front Physiol. (2018) 9:250. doi: 10.3389/fphys.2018.00250, 29643812 PMC5882847

[75] Ho CY Mealiffe ME Bach RG Bhattacharya M Choudhury L Edelberg JM . Evaluation of mavacamten in symptomatic patients with nonobstructive hypertrophic cardiomyopathy. J Am Coll Cardiol. (2020) 75:2649–60. doi: 10.1016/j.jacc.2020.03.064, 32466879

[76] Khor KH Campbell FE Owen H Shiels IA Mills PC. Myocardial collagen deposition and inflammatory cell infiltration in cats with pre-clinical hypertrophic cardiomyopathy. Vet J. (2015) 203:161–8. doi: 10.1016/j.tvjl.2014.11.018, 25573453

[77] Kitz S Fonfara S Hahn S Hetzel U Kipar A. Feline hypertrophic cardiomyopathy: the consequence of cardiomyocyte-initiated and macrophage-driven remodeling processes? Vet Pathol. (2019) 56:565–75. doi: 10.1177/0300985819837717, 30895910

[78] Novo Matos J Garcia-Canadilla P Simcock IC Hutchinson JC Dobromylskyj M Guy A . Micro-computed tomography (micro-CT) for the assessment of myocardial disarray, fibrosis and ventricular mass in a feline model of hypertrophic cardiomyopathy. Sci Rep. (2020) 10:20169. doi: 10.1038/s41598-020-76809-5, 33214588 PMC7678873

[79] Rodríguez JMM Fonfara S Hetzel U Kipar A. Feline hypertrophic cardiomyopathy: reduced microvascular density and involvement of CD34+ interstitial cells. Vet Pathol. (2022) 59:269–83. doi: 10.1177/03009858211062631, 34955067 PMC8928422

[80] Oleynikov D. Myocardial expression of connexin 43 in cats with hypertrophic and restrictive cardiomyopathy phenotype. Open Vet J. (2025) 15:1244–52. doi: 10.5455/OVJ.2025.v15.i3.16, 40276190 PMC12017731

[81] Fonfara S Kitz S Monteith G Hahn S Kipar A. Myocardial transcription of inflammatory and remodeling markers in cats with hypertrophic cardiomyopathy and systemic diseases associated with an inflammatory phenotype. Res Vet Sci. (2021) 136:484–94. doi: 10.1016/j.rvsc.2021.03.027, 33848803

[82] Tablin F Schumacher T Pombo M Marion CT Huang K Norris JW . Platelet activation in cats with hypertrophic cardiomyopathy. J Vet Intern Med. (2014) 28:411–8. doi: 10.1111/jvim.12325, 24612013 PMC4857988

[83] Schober KE Maerz I. Doppler echocardiographic assessment of left atrial appendage flow velocities in normal cats. J Vet Cardiol. (2005) 7:15–25. doi: 10.1016/j.jvc.2004.11.001, 19083314

[84] Ito T Suwa M. Assessment of left atrial appendage function by echocardiography. Heart Fail Rev. (2023) 28:1177–87. doi: 10.1007/s10741-023-10298-2, 36800057

[85] Karim N Ho SY Nicol E Li W Zemrak F Markides V . The left atrial appendage in humans: structure, physiology, and pathogenesis. Europace. (2020) 22:5–18. doi: 10.1093/europace/euz212, 31578542

[86] Grassinger JM Henrich M Echevarría AC März I Henrich E Bartel A . Correlation of histopathological changes in the left atrium and left atrial appendage with the degree of dilation in cats. J Comp Pathol. (2021) 189:8–25. doi: 10.1016/j.jcpa.2021.09.001, 34886990

[87] Beigel R. Wunderlich NC. Siew Z. Ho Y. Arsanjani R. Siegel RJ. The left atrial appendage: anatomy, function, and noninvasive evaluation. JACC: Cardiovascular Imaging. (2014) 7:1251–65. doi: 10.1016/j.jcmg.2014.08.009 25496544

[88] Kochie SL Schober KE Rhinehart J Winter RL Bonagura JD Showers A . Effects of pimobendan on left atrial transport function in cats. J Vet Intern Med. (2021) 35:10–21. doi: 10.1111/jvim.15976, 33241877 PMC7848333

[89] Lo Presti AM Monteleone A Musotto G Tamburini A Napoli E Burriesci G. Modelling of thrombus formation, growth and embolisation in the left atrial appendage under atrial fibrillation. Comput Biol Med. (2025) 191:110134. doi: 10.1016/j.compbiomed.2025.110134, 40198982

[90] Stokol T Brooks M Rush JE Rishniw M Erb H Rozanski E . Hypercoagulability in cats with cardiomyopathy. J Vet Intern Med. (2008) 22:546–52. doi: 10.1111/j.1939-1676.2008.0098.x, 18466239

[91] Gavazza A Marchegiani A Guerriero L Turinelli V Spaterna A Mangiaterra S . Updates on laboratory evaluation of feline cardiac diseases. Vet Sci. (2021) 8:41. doi: 10.3390/vetsci803 33802401 PMC8000286

[92] Jandrey KE Norris JW MacDonald KA Kittleson MD Tablin F. Platelet function in clinically healthy cats and cats with hypertrophic cardiomyopathy: analysis using the Platelet Function Analyzer-100. Vet Clin Pathol. (2008) 37:385–8. doi: 10.1111/j.1939-165X.2008.00062.x, 19055572

[93] Cremer SE Koch J Graversen N Gravgaard AS Langhorn R Kristensen AT . Analytical validation of platelet microparticle quantification in cats. Vet Clin Pathol. (2018) 47:386–95. doi: 10.1111/vcp.12641, 30199121

[94] Shaverdian M Nguyen N Li RHL. A novel technique to characterize procoagulant platelet formation and evaluate platelet procoagulant tendency in cats by flow cytometry. Front Vet Sci. (2024) 11:1480756. doi: 10.3389/fvets.2024.1480756, 39742312 PMC11685743

[95] Li RHL Fabella A Nguyen N Kaplan JL Ontiveros E Stern JA. Circulating neutrophil extracellular traps in cats with hypertrophic cardiomyopathy and cardiogenic arterial thromboembolism. J Vet Intern Med. (2023) 37:490–502. doi: 10.1111/jvim.16676, 36951591 PMC10061180

[96] Yoon LI Li RHL. Evaluation of neutrophil:lymphocyte and platelet:neutrophil ratios and their prognostic utility in cats with hypertrophic cardiomyopathy and cardiogenic arterial thromboembolism. J Feline Med Surg. (2025) 27:27. doi: 10.1177/1098612X251376773, 41165112 PMC12576060

[97] Bédard C Lanevschi-Pietersma A Dunn M. Evaluation of coagulation markers in the plasma of healthy cats and cats with asymptomatic hypertrophic cardiomyopathy. Vet Clin Pathol. (2007) 36:167–72. doi: 10.1111/j.1939-165x.2007.tb00203.x, 17523090

[98] Rubiś P Natorska J Ząbczyk M Dziewięcka E Karabinowska A Wiśniowska-Śmiałek S . Fibrin clot properties and thrombin generation in hypertrophic cardiomyopathy. Thromb Haemost. (2020) 120:181–3. doi: 10.1055/s-0039-1697956, 31659736

[99] Mcmichael MA Smith SA. Viscoelastic coagulation testing: technology, applications, and limitations. Vet Clin Pathol. (2011) 40:140–53. doi: 10.1111/j.1939-165X.2011.00302.x, 21446994

[100] Rosati T Jandrey KE Burges JW Kent MS. Establishment of a reference interval for a novel viscoelastic coagulometer and comparison with thromboelastography in healthy cats. Vet Clin Pathol. (2020) 49:660–4. doi: 10.1111/vcp.12916, 33207006

[101] Sigrist NE Schefer RJJ Kutter APN. Characteristics of hyperfibrinolysis in dogs and cats demonstrated by rotational thromboelastometry (ROTEM). Vet J. (2018) 242:67–73. doi: 10.1016/j.tvjl.2018.11.002, 30503547

[102] Döderlein E Mischke R. Reference intervals for thromboelastometry with the ROTEM® delta in cats. Res Vet Sci. (2015) 100:271–6. doi: 10.1016/j.rvsc.2015.03.005, 25910691

[103] Assunção PCF. Tromboelastometry (ROTEM) in Maine Coon breed cats carriers and no carriers of A31P mutation in the MYBPC3 gene for hipertrofic myocardiopathy. Botucatu: Universidade Estadual Paulista “Júlio de Mesquita Filho” (2019). p. 1–112.

[104] Hall DJ Rush JE deLaforcade AM Shaw SP. Kaolin-activated thromboelastography in echocardiographically normal cats. Am J Vet Res. (2012) 73:775–8. doi: 10.2460/ajvr.73.6.775, 22620690

[105] Engelen C Moritz A Barthel F Bauer N. Preliminary reference intervals and the impact of citrate storage time for thrombelastography in cats including delta and the velocity curve. BMC Vet Res. (2017) 13:366. doi: 10.1186/s12917-017-1278-y, 29187198 PMC5707899

[106] Johnson AJ Rozanski EA de Laforcade AM Davila C Rush JE Guillaumin J. Viscoelastic coagulation monitoring parameters in cats with acute arterial thromboembolism. J Vet Intern Med. (2024) 38:2045–51. doi: 10.1111/jvim.17050, 38747192 PMC11256171

[107] Langhorn R Bach MBT Gravgaard AS Graversen N Olsen CL Monrad KH . Applicability of global hemostatic tools for evaluation of Hemostatic state and detection of thrombosis in cats with cardiomyopathies. J Vet Intern Med. (2025) 39:e70250. doi: 10.1111/jvim.70250, 40976873 PMC12450611

[108] Whitton TP Healy WJ. Review of Thromboelastography (TEG): medical and surgical applications. Ther Adv Pulm Crit Care. (2023) 18:29768675231208426. doi: 10.1177/29768675231208426, 38107072 PMC10725099

[109] Selby R. “TEG talk”: expanding clinical roles for thromboelastography and rotational thromboelastometry. Hematology Am Soc Hematol Educ Program. (2020) 2020:67–75. doi: 10.1182/hematology.2020000090, 33275705 PMC7727516

[110] Broomfield J-A Abidia A Gopal J. Thromboelastography and clinical outcomes in peripheral arterial disease: a systematic review and narrative synthesis. Ann R Coll Surg Engl. (2026) 108:170–83. doi: 10.1308/rcsann.2025.0091, 41556223 PMC12949706

[111] Kim Y Patel SS McElroy IE DeCarlo C Bellomo TR Majumdar M . A systematic review of thromboelastography utilization in vascular and endovascular surgery. J Vasc Surg. (2022) 75:1107–15. doi: 10.1016/j.jvs.2021.11.037, 34788649

[112] Hincker A Feit J Sladen RN Wagener G. Rotational thromboelastometry predicts thromboembolic complications after major non-cardiac surgery. Crit Care. (2014) 18:549. doi: 10.1186/s13054-014-0549-2, 25292221 PMC4200117

[113] Sai AD Prithvishree R Savan KN Ganesh M Nitin G Asish K . Clinical utility of thromboelastography in patients with COVID-19: a prospective observational study. BMC Emerg Med. (2025) 25:227. doi: 10.1186/s12873-025-01381-y, 41214509 PMC12604182

[114] Shah A Donovan K McHugh A Pandey M Aaron L Bradbury CA . Thrombotic and haemorrhagic complications in critically ill patients with COVID-19: a multicentre observational study. Crit Care. (2020) 24:561. doi: 10.1186/s13054-020-03260-3, 32948243 PMC7499016

[115] Naguib AN Carrillo SA Corridore M Bigelow AM Walczak A Tram NK . A ROTEM-guided algorithm aimed to reduce blood product utilization during neonatal and infant cardiac surgery. J Extra Corpor Technol. (2023) 55:60–9. doi: 10.1051/ject/2023017, 37378438 PMC10304858

[116] Ito T Suwa M. Left atrial spontaneous echo contrast: relationship with clinical and echocardiographic parameters. Echo Res Pract. (2019) 6:R65–73. doi: 10.1530/ERP-18-0083, 30959476 PMC6499934

[117] Huang O Shi Z Garg N Jensen C Palmeri ML. Automated spontaneous echo contrast detection using a multisequence attention convolutional neural network HHS public access. Ultrasound Med Biol. (2024) 50:788–96. doi: 10.5281/zenodo.10795591 38461036 PMC11060922

[118] Zhang K Yu H Lu Y Zhang P Liu D Huang J . Does spontaneous Echo contrast in the left atrial appendage increase thromboembolism risk after left atrial appendage closure? A retrospective study on its impact on device-related thrombosis and arterial thromboembolic events. Cardiovasc Ther. (2025) 2025:1849432. doi: 10.1155/cdr/1849432, 40255275 PMC12008483

[119] Wang B Wang Z Fu G He B Wang H Zhuo W . Left atrial spontaneous echo contrast and ischemic stroke in patients undergoing percutaneous left atrial appendage closure. Front Cardiovasc Med. (2021) 8:723280. doi: 10.3389/fcvm.2021.723280, 34631825 PMC8495018

[120] Klein AL Daniel Murray R Black IW Facc Shalabh Chandra F Grimm RA D AP . Integrated backscatter for quantification of left atrial spontaneous echo contrast. JACC. (1996) 28:222–31. 8752818 10.1016/0735-1097(96)00131-3

[121] Lo ST Walker AL Georges CJ Li RHL Stern JA. Dual therapy with clopidogrel and rivaroxaban in cats with thromboembolic disease. J Feline Med Surg. (2022) 24:277–83. doi: 10.1177/1098612X211013736, 33966532 PMC8830184

[122] Perzborn E Roehrig S Straub A Kubitza D Mueck W Laux V. Rivaroxaban: a new oral factor Xa inhibitor. Arterioscler Thromb Vasc Biol. (2010) 30:376–81. doi: 10.1161/ATVBAHA.110.202978, 20139357

[123] Li RHL Stern JA Ho V Tablin F Harris SP. Platelet activation and clopidogrel effects on ADP-induced platelet activation in cats with or without the A31P mutation in MYBPC3. J Vet Intern Med. (2016) 30:1619–29. doi: 10.1111/jvim.14568, 27615120 PMC5032873

[124] Ho KK Abrams-Ogg ACG Wood RD O’Sullivan ML Kirby GM Blois SL. Assessment of platelet function in healthy cats in response to commonly prescribed antiplatelet drugs using three point-of-care platelet function tests. J Feline Med Surg. (2017) 19:638–47. doi: 10.1177/1098612X16648182, 27170631 PMC11128806

[125] Lo ST Li RHL Georges CJ Nguyen N Chen CK Stuhlmann C . Synergistic inhibitory effects of clopidogrel and rivaroxaban on platelet function and platelet-dependent thrombin generation in cats. J Vet Intern Med. (2023) 37:1390–400. doi: 10.1111/jvim.16727, 37208839 PMC10365033

